# Task-sharing interventions for improving control of diabetes in low-income and middle-income countries: a systematic review and meta-analysis

**DOI:** 10.1016/S2214-109X(20)30449-6

**Published:** 2020-11-23

**Authors:** Joseph Linju Maria, T N Anand, Boban Dona, Jose Prinu, Dorairaj Prabhakaran, Panniyammakal Jeemon

**Affiliations:** a Centre for Chronic Disease Control, New Delhi, India; b Public Health Foundation of India, New Delhi, India; c Achutha Menon Centre for Health Science Studies, Sree Chitra Tirunal Institute for Medical Sciences and Technology, Trivandrum, India

## Abstract

**Background:**

Task-sharing interventions using non-physician health-care workers might be a potential diabetes management strategy in health systems that are constrained by physician shortages, such as those in low-income and middle-income countries (LMICs).

**Methods:**

We did a systematic review and meta-analysis of task-sharing intervention strategies for managing type 2 diabetes in LMICs. We searched PubMed, Embase, and CINAHL from database inception to Sept 25, 2019, for studies that were randomised control trials or cluster randomised trials with task-shifted or task-shared interventions delivered to adults (≥18 years) by non-physician health workers versus usual care, done in LMICs with glycated haemoglobin (HbA_1c_) or fasting blood sugar (FBS) as outcome measures. The methodological quality of included studies was assessed using the Cochrane risk of bias tool. Random-effects model meta-analysis was used to estimate the population average pooled mean difference for HbA_1c_ and FBS with 95% CIs. Our study protocol was registered in the PROSPERO database (CRD42018081015).

**Findings:**

We found 4213 studies from the literature search, of which 46 (1·1%) were eligible for the narrative synthesis, including a total of 16 973 participants. 16 of these studies were excluded from the meta-analysis due to high risk of bias. 24 studies with a total of 5345 participants were included in the meta-analysis of HbA_1c_ and 18 studies with a total of 3287 participants for FBS. Interventions led to an average reduction in HbA_1c_ when tasks were delivered by nurses (averaged pooled mean difference −0·54% [95% CI −0·89 to −0·18]; *I*^2^=80%) and pharmacists (−0·91% [–1·15 to −0·68]; *I*^2^=58%), but not when they were delivered by dietitians (−0·50% [–1·10 to 0·09]; *I*^2^=54%) or community health workers (0·05% [0·03 to 0·07]; *I*^2^=0%). A reduction in average FBS was also observed when interventions were delivered by pharmacists (average pooled mean difference −36·26 mg/dL [–52·60 to −19·92]; *I*^2^=78%) but not nurses (−7·46 mg/dL [–18·44 to 3·52]; *I*^2^=79%) or community health workers (−5·41 [–12·74 to 1·92]; *I*^2^=71%). Only one study reported on FBS when tasks were delivered by dietitians, with a mean difference of −35·00 mg/dL (−65·96 to −4·04).

**Interpretation:**

Task sharing interventions with non-physician healthcare workers show moderate effectiveness in diabetes management in LMIC settings. Although relatively high heterogeneity limits the interpretation of the overall findings, interventions led by pharmacists and nurses in LMICs with relatively high physician density are effective strategies in the management of diabetes.

**Funding:**

Wellcome Trust–Department of Biotechnology India Alliance.

## Introduction

Non-communicable diseases (NCDs) continue to be a dominant cause of global deaths. For example, the Global Burden of Disease study^1^ estimates that NCDs contributed up to three-quarters of total annual deaths in 2019. Furthermore, low-income and middle-income countries (LMICs) bear a disproportionately higher burden of NCDs than high-income countries. Diabetes is a major contributor to NCDs, affecting about 463 million people globally. Consequent to poor detection rates and awareness of diabetes, four of five adults with undiagnosed diabetes live in LMICs. Additionally, the treatment and control rates of diabetes are abysmally poor in LMICs.^2^ It has been estimated that the total unmet need for diabetes care in terms of poor detection, treatment, and control is about 77% in LMIC settings.^2^, ^3^

The management of diabetes is complex, necessitating continuous effort for achieving better control and evidence-based targets.^4^ The American Medical Association recommends team-based, patient-centred care, integrated long-term treatment approaches to diabetes and comorbidities, and ongoing collaborative communication among providers for improving diabetes care and population health as per the standards of medical care in diabetes.^5^, ^6^ Further, the management of diabetes becomes more multifaceted with other concurrent chronic conditions. Data suggest that as many as 85% of people with diabetes have at least one other chronic condition.^7^ Complications of poor glycaemic control, such as macrovascular diseases, blindness, and kidney failure, are often devastating for the individual and family, and incur catastrophic health spending in LMIC settings.^8^, ^9^ There is a clear shortage of well trained health-care providers in LMICs, despite the increased demand for integrated care and the emphasis on the availability of trained personnel at decentralised levels of health care to provide integrated care for diabetes.^10^, ^11^ The average physician consultation time is often low in LMICs, which leads to ineffective communication, clinical handover, and lifestyle counselling for patients with diabetes.^12^

**Research in context**

**Evidence before this study**

Task-sharing has been proposed as a strategy to deal with the shortage of physicians in low-income and middle-income countries (LMICs). We searched PubMed, Embase, and CINAHL for task-sharing interventions in managing people with diabetes. Two previous reviews show that task sharing for managing non-communicable diseases is a potentially viable strategy, and another review reported reductions in population average blood pressure with task-sharing strategies. However, no systematic reviews or meta-analyses of task-sharing interventions for managing diabetes have been published.

**Added value of this study**

In this systematic review and meta-analysis, we determined the effectiveness of task-sharing interventions for managing diabetes in LMICs. We showed that task-sharing interventions led by nurses and pharmacists are effective in achieving meaningful population average reductions in glycated haemoglobin and fasting blood sugar. However, task-sharing interventions with community health workers did not show meaningful reductions. Additionally, the task-sharing strategy was more effective in LMICs with higher doctor–population ratios.

**Implications of all the available evidence**

Our data provide evidence to support task-sharing interventions that involved higher cadres of health-care providers, such as nurses and pharmacists, in managing diabetes in LMIC settings. The magnitude of the population-average reduction in glycated haemoglobin and fasting blood sugar are similar to the reductions associated with oral hypoglycaemic drugs in clinical trials. The supervision and levels of training needed for the health-care workforce should be determined and tailored according to the capacity of the health system.

Reorganising health service delivery using non-physician health-care workers has been successful in improving outcomes for maternal and child health care, and a similar reorganising approach might improve outcomes of diabetes care. Task shifting,^13^ an approach for redistributing human resources rationally, could help in improving health-care delivery.^14^ In general, task-shifting describes a situation in which a task that is normally performed by a physician is transferred to a health professional with a different or lower level of education and training. This approach can also include lower cadres of health-care professionals being trained in a particular task.^15^, ^16^ The term task sharing has been used recently to describe this concept, as it better describes the process of care in team-based provision of integrated care for patients with chronic conditions.^17^ Previous systematic reviews highlight task sharing as a potential strategy for managing NCDs.^13^, ^18^ We examined the nature and effectiveness of task sharing interventions for managing diabetes in LMIC settings.

## Methods

### Search strategy and selection criteria

For this systematic review and meta-analysis, we did a systematic search to summarise task-sharing interventions for managing diabetes. We developed a search strategy in PubMed using previous reviews,^18^ which we modified for use in other databases (Embase and CINAHL) for locating articles from inception to Dec 28, 2018. We then did an updated search for articles published before Sept 25, 2019. No language restrictions were used. A range of search terms was used, relating to diabetes, task sharing, task shifting, and a list of LMICs based on the 2018 World Bank database^19^ (appendix pp 41–45). Hand searching was done using citations and reference lists of the studies included.

We included randomised control trials or cluster randomised trials with task-shifted or task-shared interventions versus usual care that were done in LMICs. To be included, trials had to be of patients aged 18 years or older with type 2 diabetes and had to involve measurement and reporting of glycaemic outcomes as the change in glycated haemoglobin or fasting blood glucose. Task shared interventions had to be designed to improve glycaemic control and to be delivered by community health workers, nurses, pharmacists, or allied health professionals (eg, dietitians). The tasks shared included non-pharmacological measures (eg, patient education for lifestyle modification) and pharmacological measures (eg, initiation or refill of prescription medications and titrating the dose of medications). We excluded studies of children, mothers with gestational diabetes, or patients with type 1 diabetes, and studies that did not involve task-sharing interventions for diabetes or cardiovascular risk management. Interventions led by peer educators or home carers were also excluded, because they are not health-care professionals, and studies of task-sharing activities that are exclusive to traditional healers or alternative therapies (eg, acupuncture and homoeopathic medicine), or that promoted only self-care or informal caregiver health education. Additionally, we excluded reviews, pre-post studies, cross-sectional studies, case-control studies, case series studies, and drug efficacy studies, as well as studies that had a duration of less than 3 months and studies that measured knowledge and attitude or practice outcomes without reported glycaemic measures.

Two authors (JLM and TNA) did the literature search independently, using the search strategy developed in consultation with the other authors. Two authors (JLM and BD) independently removed the duplicates manually. Further, they reviewed all the titles independently, and any conflicts in article selection were resolved after mutual discussion (between JLM and BD). The remaining abstracts were assessed for potential eligibility by the same reviewers (JLM and BD). Finally, three authors (JLM, BD, and JP) independently reviewed the full texts of the included articles, and any disagreements were resolved through discussion with a fourth reviewer (PJ). Our study protocol was registered in the PROSPERO database (CRD42018081015).

### Data analysis

Studies that met the inclusion criteria after the full-text review were assessed for quality by two authors (JLM and BD) using the Cochrane risk of bias tool.^20^ Double data extraction was done by two authors (BD and JP) from the eligible full articles. Any discrepancies were discussed and clarified with two other authors (JLM and TNA). The arbitrator (PJ) reviewed any apparent discrepancies and made the final recommendation. We extracted the details of patients, the delivered interventions, components of the intervention, and relevant results of the studies. We extracted the outcome measurements for glycated haemoglobin (HbA_1c_) and fasting blood glucose (FBS) that were taken before and after the intervention, for both the intervention and control groups. Information regarding the country where the study was conducted and study population type and size were also noted.

We did a qualitative synthesis of types of intervention and sample characteristics of the included trials. For quantitative synthesis, we included eligible randomised controlled trials and clustered randomised trials with at least 30 participants in each treatment arm for our meta-analysis. For cluster randomised trials, we estimated the effective study sample size using the reported design effect, or we calculated the design effect on the basis of the intra-cluster correlation coefficient and average cluster size. A change in glycaemic levels was estimated using the difference between the mean HbA_1c_ or FBS in the intervention arm (ie, task sharing) and the control group (ie, usual care).

We adopted a random effect model for meta-analysis because we assumed a greater study-level variability due to differences in task-sharing groups, types of interventions, and study populations.^21^, ^22^ Appropriate weights were assigned for individual studies included in the meta-analysis on the basis of the inverse variance method. The Der Simonian and Laird method was used for assessing between-study variance.^23^ We estimated the pooled mean difference for HbA_1c_ and FBS along with their 95% CIs. We generated prediction intervals to assess the uncertainty of the summary estimate across different study settings.^21^, ^24^ We conducted independent heterogeneity assessments for HbA_1c_ and FBS analyses. Heterogeneity was assessed using the *I*^2^ statistic, and the statistical significance of heterogeneity was tested with Q statistics.^25^ The heterogeneity contribution from each study was assessed by omitting each study and recording the change in overall heterogeneity.

An exploratory subgroup analysis was done with the covariates sample size, duration of intervention, country physician density, study population characteristics, and geographical regions as classified by WHO. We did univariate and multivariate meta-regression analyses to identify the effect of covariates on the effect size.^26^ Publication bias was graphically assessed with funnel plots and contour-enhanced funnel plots.^27^ We also used Egger's regression test^28^ for the statistical significance of publication bias. We analysed data using the meta package of R version 3.5.1.^29^ The quality of evidence was evaluated using GRADE.^30^ Ethics approval was not required for this study.

### Role of the funding source

The funder had no role in the study design, data collection, data analysis, data interpretation, or the writing of the report. All authors had full access to all the data in the study and had final responsibility for the decision to submit for publication.

## Results

**Figure 1:**
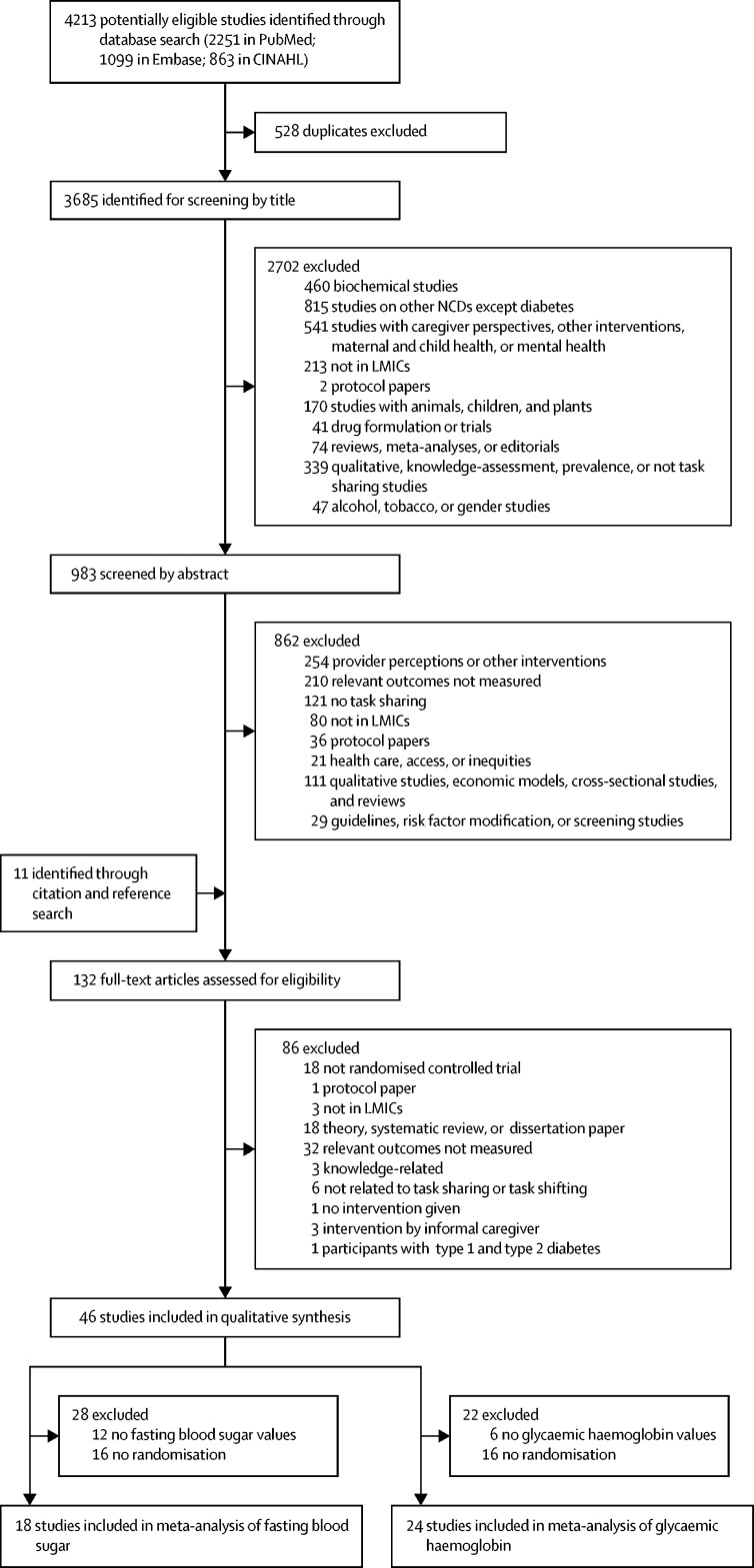
Study selection NCDs=non-communicable diseases. LMICs=low-income and middle-income countries.

We identified 4213 references from all the databases (figure 1), 528 (12·5%) of which were duplicates. We screened 3685 (87·5%) of the titles and 983 (23·3%) abstracts for eligibility, and promoted 121 (2·9%) articles for full-text review. Additionally, 11 studies were obtained after hand searching and were also included in the full-text review. Of the 132 articles selected for full-text review, 86 (65·2%) were excluded, most commonly because the relevant outcomes were not measured, and 46 (34·8%) were included in the narrative synthesis, including a total of 16 973 participants (figure 1).

The characteristics of the included studies are summarised in the appendix (pp 2–16). Among the 46 included studies, there were eight (17%) cluster randomised trials^31^, ^32^, ^33^, ^34^, ^35^, ^36^, ^37^, ^38^ and 38 (83%) randomised trials.^39^, ^40^, ^41^, ^42^, ^43^, ^44^, ^45^, ^46^, ^47^, ^48^, ^49^, ^50^, ^51^, ^52^, ^53^, ^54^, ^55^, ^56^, ^57^, ^58^, ^59^, ^60^, ^61^, ^62^, ^63^, ^64^, ^65^, ^66^, ^67^, ^68^, ^69^, ^70^, ^71^, ^72^, ^73^, ^74^, ^75^, ^76^ There were nine trials each from Brazil and China. The studies were done in primary health centres (n=10 [22%]),^31^, ^33^, ^35^, ^50^, ^63^, ^65^, ^66^, ^70^, ^72^, ^74^ diabetes clinics or community health centres (n=12 [26%]),^32^, ^34^, ^36^, ^45^, ^47^, ^48^, ^49^, ^51^, ^67^, ^68^, ^71^, ^75^ hospitals (n=21 [44%]),^39^, ^40^, ^41^, ^42^, ^43^, ^44^, ^46^, ^52^, ^53^, ^55^, ^56^, ^57^, ^58^, ^59^, ^60^, ^61^, ^62^, ^64^, ^69^, ^73^, ^76^ and community settings (n=3 [9%]).^37^, ^38^, ^54^ The sample size ranged from 53 participants^65^ to 4393 participants.^34^ 34 (74%) of 46 studies were with patients with diabetes. The other studies were with older patients (n=1 [2%]),^51^ patients with high risk of diabetes (n=2 [4%]),^48^, ^74^ or patients with cardiovascular disease (n=1 [2%]),^37^ hypertension (n=1 [2%]),^76^ hypertension and diabetes (n=2 [4%]),^38^, ^66^ metabolic syndrome (n=1 [2%]),^44^ coronary artery disease (n=2 [4%]),^46^, ^49^ myocardial infarction (n=1 [2%]),^43^ or NCD (n=1 [2%]).^33^ The participant follow-up ranged from 2 months^42^, ^43^ to 36 months.^66^, ^71^ 21 (46%) of 46 trials followed up participants for 12 months or more.

On the basis of the strategies used as the predominant component, interventions led by non-physician health-care workers were broadly categorised as pharmacological or non-pharmacological. However, there were multiple elements in the interventions of the studies included. Pharmacological interventions involved the generation of a medication prescription following an algorithm or guidelines, dose adjustment or titration of medications, and drug modification. Non-pharmacological interventions included assessment, monitoring, lifestyle education, and counselling for the management of diabetes. The interventions were delivered by pharmacists (n=16 [35%]),^41^, ^42^, ^44^, ^45^, ^52^, ^53^, ^54^, ^55^, ^56^, ^61^, ^62^, ^66^, ^68^, ^69^, ^70^, ^73^ nurses (n=19 [41%]),^31^, ^33^, ^34^, ^35^, ^36^, ^39^, ^40^, ^43^, ^46^, ^49^, ^57^, ^59^, ^64^, ^65^, ^67^, ^71^, ^72^, ^75^, ^77^ dietitians or nutritionists (n=4 [9%]),^47^, ^50^, ^58^, ^74^ community health workers (n=5 [11%]),^32^, ^37^, ^38^, ^48^, ^63^ or a combination of community health centre staff, nurses, or pharmacists (n=2 [4%]).^51^, ^60^

Of the 46 trials, 37 (80%) delivered a non-pharmacological intervention, eight (17%) had both non-pharmacological and pharmacological components,^38^, ^40^, ^42^, ^44^, ^52^, ^61^, ^66^, ^69^ and one (2%)^33^ described a pharmacological intervention exclusively. Pharmacological components in the trials ranged from drug initiation (n=3 [7%])^33^, ^44^, ^66^ to dose adjustment (n=8 [17%])^38^, ^40^, ^42^, ^44^, ^52^, ^61^, ^66^, ^69^ and the addition of new drugs (n=2 [4%]).^33^, ^69^ The pharmacological interventions were task-shared with pharmacists (n=6 [13%]),^42^, ^44^, ^52^, ^61^, ^66^, ^69^ nurses (n=2 [4%]),^33^, ^40^ and community health workers (n=1 [2%]).^38^ The non-pharmacological interventions described in the trials ranged from providing education regarding lifestyle modification (n=45 [98%]) to telephone follow-up (n=12 [26%])^39^, ^40^, ^41^, ^42^, ^46^, ^52^, ^54^, ^55^, ^57^, ^60^, ^71^, ^72^ and home visits (n=5 [11%]).^49^, ^63^, ^64^, ^67^, ^75^ The contents of lifestyle education were focused on dietary changes (n=39 [85%]), physical activity (n=38 [83%]), and medication adherence (n=37 [80%]).

19 (41%) of the interventions were organised as individual sessions, 14 (30%) had group sessions, and seven (15%) had a mixture of both group and individual sessions. The format of intervention sessions was not described in six (13%) studies. The frequency of sessions with the participants in the intervention group varied. Some studies had weekly sessions and monthly follow-up classes, whereas others had one class per month for up to 6 months.

The detailed results of the quality assessment based on Cochrane risk of bias tool are presented in the appendix (pp 19–20). Of the 46 trials, 16 (35%) studies^31^, ^37^, ^41^, ^49^, ^53^, ^55^, ^58^, ^60^, ^61^, ^62^, ^64^, ^67^, ^68^, ^69^, ^73^, ^74^ did not sufficiently describe randomisation, or the method of randomisation was unclear. Consequently, those trials were considered to have a high risk of bias and were not included in the meta-analysis. Allocation concealment was reported only in 14 (30%) studies,^31^, ^32^, ^36^, ^38^, ^39^, ^43^, ^46^, ^47^, ^51^, ^54^, ^63^, ^65^, ^71^, ^72^ whereas 19 (41%) studies^32^, ^34^, ^36^, ^37^, ^38^, ^39^, ^40^, ^41^, ^43^, ^44^, ^45^, ^46^, ^47^, ^57^, ^63^, ^68^, ^69^, ^70^, ^72^ reported masking of outcome assessors.

In terms of the availability of outcome variables, of the 30 trials considered for meta-analyses, six did not report before and after values for HbA_1c_. Therefore, 24 studies were included in the meta-analysis of the HbA_1c_ outcome. Similarly, 12 studies did not have before and after values for FBS, and therefore 18 studies were included in the meta-analysis of the FBS outcome.

**Figure 2:**
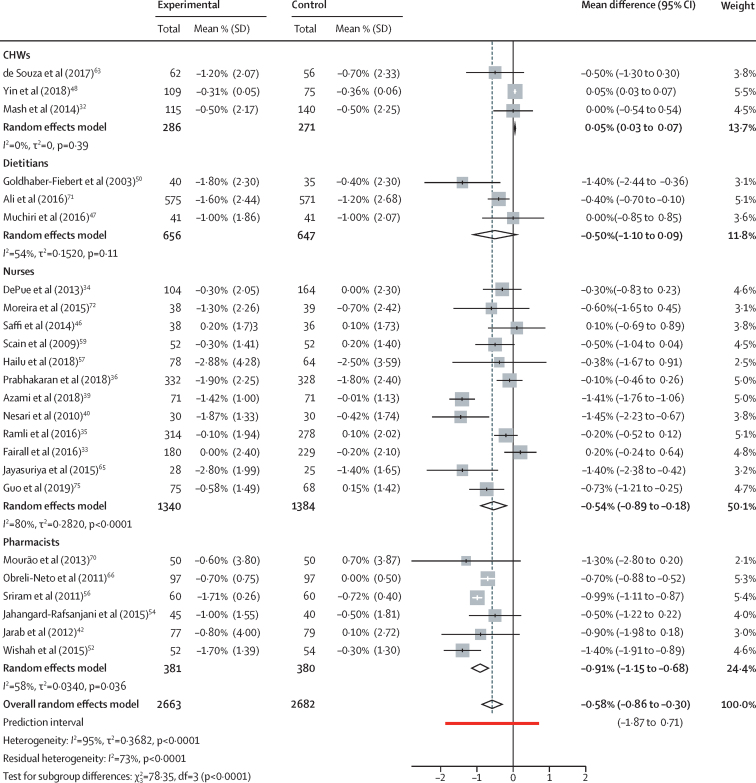
Effect of task-sharing interventions for diabetes control on glycated haemoglobin CHWs=community health workers.

Regarding the effect of interventions on HbA_1c_, we included 24 studies (19 randomised controlled trials and five cluster randomised trials) in the analysis after assessing risk of bias. Overall, the population average pooled mean difference in HbA_1c_ was −0·58% (95% CI −0·86 to −0·30; *I*^2^=95%) with a prediction interval ranging from −1·87 to 0·71 (figure 2). Simple funnel plots for publication bias showed asymmetry (appendix p 27), and the Eggers regression test also reported significant bias (t=–3·41; df=22; p=0·0024). To assess the reason for the asymmetric funnel plot, we examined contour-enhanced funnel plots. Most of the missing studies were in the areas of high statistical significance and unlikely to be due to publication bias (appendix p 27).

In the pooled effects on HbA_1c_, studies involving task sharing with community health workers showed an averaged pooled mean difference of 0·05% (95% CI 0·03 to 0·07), whereas for dietitians it was −0·50% (−1·10 to 0·09) and for nurses it was −0·54% (−0·89 to −0·18; figure 2). In the subgroup analysis, for nurses implementing non-pharmacological interventions the averaged pooled mean difference was −0.54% (−0·91 to −0·18), compared with −0·59% (−2·21 to 1·02) for them implementing pharmacological interventions (appendix p 28). Task sharing with pharmacists showed an average pooled mean difference of −0·91% (−1·15 to −0·68), and pharmacologists implementing both pharmacological and non-pharmacological interventions showed similar effects on HbA_1c_ (figure 2; appendix p 28).

In subgroup analysis of the effects on HbA_1c_, task-sharing interventions in the diabetes population showed an average pooled mean difference of −0·68% (95% CI −0·92 to −0·44; appendix p 29), and restriction to sample sizes of more than 200 showed an average pooled mean difference of −0·17% (−0·34 to 0·00; appendix p 30). Subgroup analysis with different intervention time periods showed an average pooled mean difference in HbA_1c_ of −0·45% (−0·78 to −0·12) for studies of 6–15 months and −0·57% (−0·86 to −0·28) for studies of more than 15 months (appendix p 31). The average pooled mean difference in study settings with up to ten doctors per 10 000 population was −0·55 (−0·89 to 0·21), and in settings with more than 15 doctors per 10 000 population it was −0·71% (−1·01 to −0·41; appendix p 32). When studies were grouped by WHO geographical regions, the average pooled mean difference in HbA_1c_ were −0·24% (−0·57 to 0·09) for the western Pacific region, −0·63% (−0·86 to −0·41) for the Americas, 0·08% (−0·23 to 0·39) in the African region, −0·64% (−1·16 to −0·13) for the southeast Asia region, and −1·23% (−1·56 to −0·90) for the eastern Mediterranean region (appendix p 33).

We assessed the contribution to heterogeneity by doing sensitivity analysis by excluding each study (appendix p 21). The exclusion of two studies^48^, ^56^ with a high heterogeneity contribution from the meta-analysis reduced the overall heterogeneity moderately, whereas the pooled estimate remained similar (−0·57 [95% CI −0·80 to −0·35]; data not shown). We identified WHO regions, study population, duration of the task-sharing intervention, and trial sample size as major predictors of effect size based on multivariate meta-regression analysis, and these covariates together accounted for 93·4% of the heterogeneity (appendix p 22).

**Figure 3:**
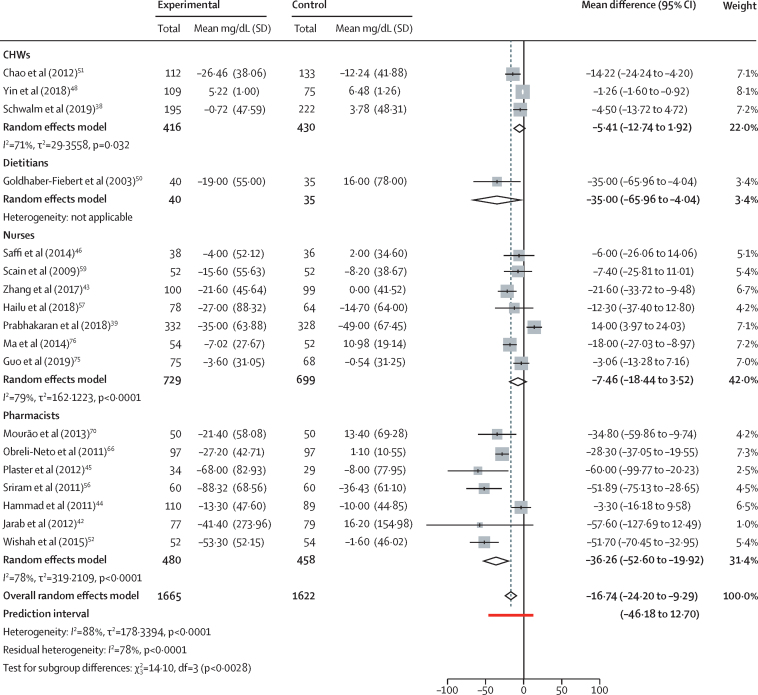
Effect of task-sharing interventions for diabetes control on fasting blood sugar CHWs=community health workers.

Concerning the effect of interventions on FBS, we included 18 individual and cluster randomised controlled trials in the analysis with 3635 participants. The overall population average pooled mean difference of FBS was −16·74 mg/dL (95% CI −24·20 to −9·29) with a prediction interval ranging from −46·18 mg/dL to 12·70 mg/d (figure 3). Funnel plots for publication bias showed asymmetry (appendix p 34) and the Eggers regression test reported significant bias (t=–3.84, df=16, p=0·0014). Visual examination of the contour-enhanced funnel plot also suggested bias, with few studies in the high significance area and missing studies in the non-significance area (appendix p 34).

We assessed mean difference in FBS when different groups implement task sharing, and it was statistically different across the groups (figure 3). Studies with community health workers showed an average pooled mean difference of −5·41 mg/dL (95% CI −12·74 to 1·92; figure 3). Task sharing with dietitians was reported in only one study^50^ with a mean difference of −35·00 mg/dL (65·96 to −4·04; figure 3). Task sharing with nurses gave an average pooled mean difference of −7·46 mg/dL (−18·44 to 3·52), and for pharmacists it was −36·26 mg/dL (−52·60 to −19·92; figure 3).

For the subgroup analysis of FBS, the effects of task sharing with pharmacological and non-pharmacological interventions are in the appendix (p 35)). Task-sharing interventions in the diabetes population showed an average pooled mean difference of −23·94 mg/dL (95% CI −38·51 to −9·37) for people with type 2 diabetes (appendix p 36), and for sample sizes with more than 200 participants the average pooled mean difference was −1·59 mg/dL (−17·40 to 14·21; appendix p 37). Subgroup analysis with different intervention time periods showed an average pooled mean difference in FBS of −35·00 mg/dL (−66·96 to −4·04) for up to 5 months, −14.86 mg/dL (−22·69 to −7·69) for 6–15 months, and −21·48 mg/dL (−35·27 to −7·69) for more than 15 months (appendix p 38). The average pooled mean difference in study settings with up to ten doctors per 10 000 population was −15·82 mg/dL (−55·60 to 24·97), and for more than 15 doctors per 10 000 population it was −25·60 mg/dL (−39·76 to −11·43; appendix p 39). The average pooled mean difference in FBS for the different WHO regions were −9·72 mg/dL (−17·22 to −2·21) for the western Pacific region, −24.34 mg/dL (−37·11 to −11·56) for the Americas, and −32·85 mg/dL (−74·13 to 8·43) for the eastern Mediterranean region (appendix p 40). We assessed the contribution to heterogeneity by doing sensitivity analysis by excluding each study (appendix p 23). In the multivariate meta-regression, the task-sharing group and sample size were significant predictors of FBS change, and these covariates together accounted for 36·8% of heterogeneity (appendix pp 23–24).

Based on the GRADE criteria, the effect of nurse-led interventions on population average reduction in HbA_1c_ was graded as moderate (appendix p 25) and was downgraded for inconsistency. Due to indirectness and inconsistency in the relationship, the level of evidence from pharmacist-led interventions were also downgraded to moderate. The evidence for lowering HbA_1c_ and FBS was rated as low for interventions delivered by community health workers.

## Discussion

We did a comprehensive review of available literature on task-sharing interventions and quantitatively synthesised the population-average pooled mean difference for HbA_1c_ and FBS levels. Our findings support the use of task-sharing interventions for the management of diabetes, with a moderate to good effect on the reduction in HbA_1c_ and FBS. However, the analyses with prediction intervals suggests that the effect of interventions on glycaemic outcomes might vary across study settings. In the subgroup analysis, the available evidence supports the engagement of higher cadres of non-physician health-care workers, such as nurses and pharmacists, for task-sharing activities related to management of diabetes. For example, non-pharmacological interventions by nurses and both non-pharmacological and pharmacological interventions by pharmacists resulted in clinically meaningful reductions in HbA_1c_. Additionally, non-pharmacological interventions delivered by pharmacists resulted in a large pooled average mean difference in FBS.

The moderate reduction in HbA_1c_ of 0·58%, as observed in our overall pooled estimate for task-sharing interventions, might lead to significant public health effects at the population level in LMIC settings. For example, a 1% reduction in HbA_1c_ was associated with a 37% reduction in microvascular complications in the UKPDS study.^77^ Similar glycaemic efficiency is reported when using dipeptidyl peptidase-4 inhibitors or gliptins, with a reduction of 0·5–1% when used as monotherapy and 0·6–1·1% when used in combination with metformin, depending on the drug, dose of therapy, and starting HbA_1c_.^78^ A systematic review of general diabetes disease management programmes also show HbA_1c_ reduction at a similar magnitude to that of our pooled analysis.^79^

Task-shared interventions for diabetes management mainly comprise non-pharmacological lifestyle interventions and pharmacological components. When delivered by higher cadres of health workers, both the non-pharmacological and pharmacological interventions resulted in meaningful reductions in HbA_1c_. In general, diabetes self-management education and support have been proven to be effective in glycaemic control.^80^, ^81^ Additionally, diabetes self-management education programmes have been organised in high-income settings using health-care workers such as nurses, pharmacists, and certified diabetes educators.^6^ Health systems in LMICs have traditionally been organised around vertical disease management programmes and hence the care processes have mostly centred on physicians. This places a high work burden on physicians and poorly defines the roles of other health-care workers in LMICs.^82^ Therefore, it is necessary to restructure and organise roles for non-physician health-care workers and to provide adequate training and supervision for effectively managing diabetes and other chronic non-communicable disease conditions in an integrated team-based care model.

The non-pharmacological and pharmacological interventions delivered by pharmacists in the studies included emphasised medication adherence as a key strategy that resulted in better glycaemic outcomes. Similar effects of glycaemic control were shown in studies that emphasised improving adherence to glycaemic medications.^83^, ^84^ Hence, adherence improvement should be considered as a major component of task sharing intervention studies in management of diabetes outcomes in LMICs.

The wide prediction intervals of the effect estimates observed in our study could be attributable to the variation in glycaemic outcomes of task-sharing strategies across different WHO regions. The WHO regions differ in terms of health-care system characteristics and doctor–population ratios. Further, they employ different cadres of health workers for task-sharing interventions. Therefore, careful selection of the cadre of health workers and the capacity for supervision by physicians are important parameters in the scale-up of task-sharing interventions to improve glycaemic outcomes in LMICs.

The implications of our study for future research and practice are that public health interventions in diabetes management that are effective and useful in selected study settings need to be scaled up and implemented more widely to create wider health impacts. However, for effective task-sharing implementation, it is necessary to strengthen health systems and have health-care regulations in terms of policies supporting non-physician health-care workers. Task-sharing of interventions in diabetes management is an ideal candidate for scaling up as a general strategy for prevention and control of NCDs in LMICs. However, as recommended in the 2019 standards of medical care in diabetes,^5^ it is important to evaluate quality improvement strategies by incorporating reliable data metrics, ongoing data collection, and evaluation with the larger aim of improving processes of care and outcomes within the available resources. A recent systematic review^85^ identifies several health system factors that support and impair the ability of non-physician health-care workers to manage NCDs. Adequate and standardised training sessions, proper guidance, reliable systems to track patient data, reasonable compensation or performance incentives for the work done, and logistical support are recognised as key facilitators to implement task-sharing interventions.^85^ Given the relative advantage of technology-assisted diabetes prevention and management interventions, more such options should be explored to expand the scope and coverage of task-sharing interventions.

The strengths of this review include a registered protocol and a comprehensive search strategy in multiple databases. This study also has limitations. First, the weak description of the intervention strategy in most studies included did not allow analysis of the effect of different types of interventions on glycaemic control. Second, significant heterogeneity was observed across the studies. However, to some extent, heterogeneity is inevitable in reviews of findings from many countries and public health interventions. This does not negate the applicability of task-shared interventions because the original populations, settings, and interventions could be quite diverse, increasing the likelihood that the evidence can be applied broadly.

In conclusion, clinicians and policy makers, when considering the organisation of care for patients with diabetes in LMICs, should consider task-sharing interventions with non-physician health-care workers. To achieve the optimal control of diabetes, non-pharmacological interventions for medication and lifestyle adherence are also needed. Prioritising potential interventions on the basis of health-care workers' availability and skills could improve glycaemic control in individuals with diabetes.

## Data sharing

All datasets generated and analysed are available in the article and appendix.
