# Supplementary material for: Task-sharing interventions for improving control of diabetes in low-income and middle-income countries: a systematic review and meta-analysis
Source: Lancet Glob Health. 2020 Nov 23;9(2):e170–80. doi: 10.1016/S2214-109X(20)30449-6 (PMC8279953; doi:10.1016/S2214-109X(20)30449-6)
Supplement: Supplementary appendix [file mmc1.pdf]

THE LANCET
Global Health

Supplementary appendix

This appendix formed part of the original submission and has been peer reviewed. We post it as supplied by the authors.

Supplement to: Maria JL, Anand TN, Dona B, Prinu J, Prabhakaran D, Jeemon P. Task-sharing interventions for improving control of diabetes in low-income and middle-income countries: a systematic review and meta-analysis. *Lancet Glob Health* 2020; published online Nov 23. http://dx.doi.org/10.1016/S2214-109X(20)30449-6.

Online supplement

Task-sharing interventions for improving control of diabetes in low-income and middle-income countries: a systematic review and meta-analysis

Corresponding author and address:

Panniyammakal Jeemon

Achutha Menon Centre for Health Science Studies

Sree Chitra Tirunal Institute for Medical Sciences and Technology

Trivandrum, Kerala, India, 695011

Email: pjeemon@gmail.com

**Table S1: Characteristics of included trials in the review**							
**Author, year (Country)**	**Sample size, Population**	**Study design**	**Task delivered by**	**Intervention**	**Follow-up**	**Outcomes assessed**	**Relevant Results**
Labhardt ,2011 (Cameroon)	221, Patients with diabetes mellitus	Cluster RCT	Nurse	1)Non pharmacologic-Counselling<br>2)Assessment of patient's motivation<br>3)Identification of psychosocial conditions<br>4) Signing of formal contract	12 months	1) Changes in Blood Pressure(BP)<br>2) Fasting Plasma Glucose(FPG) values	No significant differences in FPG values in the three study groups. Follow up data available for very few patients (n=15). Among those, 11 (73%) had a fasting plasma glucose <7 mm at the last consultation. Overall, fasting plasma glucose decreased from 10.8 (SD ± 5.2) mm at treatment start to 6.2 (±1.7) mm at the last recorded consultation (95% CI: 1.7–7.6, P < 0.01).
Mash, 2014 (South Africa)	866, Patients with diabetes mellitus	Cluster RCT	CHW (Health promoters)	1) Non pharmacologic-Group education session	12months	1)5% weight loss, and a 1% reduction in HbA1c level<br>2)Mean BP<br>3) Mean weight loss<br>4) Mean HbA1c<br>5) Mean total cholesterol levels<br>6) Mean waist circumference	No significant difference between the intervention and control groups in reduction of HbA1c levels by 1% as set by the study.
Fairall, 2016 (South Africa)	4393 (HbA1c of 704 only), Patients with one or more of	Cluster RCT	Nurse	1)Pharmacologic-Medication prescription	12 months	1)Treatment intensification<br>2)Disaggregation of primary	No significant difference in treatment intensification rates between intervention and control group for any of the four disease cohorts (Diabetes, Hypertension, Chronic

	the following: hypertension, diabetes, chronic respiratory disease, or depression.					outcomes by type of medication<br>3)Cardiovascular disease risk and risk factors such as BP, Body Mass Index (BMI)<br>4)Smoking status<br>5)Health-related quality of life<br>6)Mortality<br>7)Healthcare utilisation	respiratory disease, Depression).Health status outcomes also did not differ between the intervention and control groups.
De Pue, 2013 (American Samoa)	268, Patients with diabetes mellitus	Cluster RCT	Nurse care manager, Community Health Worker (CHW)	1)Non pharmacologic-education<br>2)Maintain appointments<br>3)Feedback to physicians about patient care needs	12 months	1)HbA1c<br>2) BMI<br>3) Waist circumference<br>4)BP	Significant reduction of HbA1c at end of treatment in intervention group when adjusting for baseline HbA1c levels, clustering and potential confounders. (b= -0.53;SE=0.21;P=0.03)
Ramli, 2016 (Malaysia)	888, Patients with diabetes mellitus	Cluster RCT	Medical assistant or nurse, pharmacist	1)Non pharmacologic-Education and counselling	12 months	1) Change in the proportion of patients achieving glycaemic target of HbA1c < 6.5%.<br>2) Change in the proportions of patients achieving the following targets:<br>1.1 BP ≤ 130/80 mmHg;<br>1.2 BMI < 23 kg/m2;<br>1.3 Waist Circumference (WC) < 90 cm for men,	Significant reduction in mean HbA1c in intervention group who received EMPOWER-PAR compared to control group (intervention:−0.1%, SE ± 0.06 vs. control: 0.2% SE ± 0.09, P = 0.003)

						< 80 cm for women<br>1.4 Total cholesterol (TC) ≤ 4.5 mmol/L<br>1.5 Triglycerides (TG) ≤ 1.7 mmol/L<br>1.6 Low density lipoprotein cholesterol (LDL-c) ≤2.6 mmol/L;<br>1.7 High density lipoprotein cholesterol (HDL-c) ≥1.1 mmol/L	
Prabhakaran, 2018<br>(India)	3698, Patients with diabetes mellitus	Cluster RCT	NCD nurse	1)Non pharmacologic-education<br>2)Lifestyle advice<br>3) Entry of patient records	12 Months	1)Between group differences in mean change from baseline to 1 year in SBP and HbA1c among participants with Hypertension and DM respectively<br>2)Between group difference in mean change in FPG, total cholesterol, BMI	No significant difference in the change in HbA1c and FPG between the two groups.

Khetan, 2019	1242 Participants who had at least 1 risk factor (hypertension, diabetes, smoking)	Cluster RCT	Community Health Workers	1)Non-pharmacologic- Home based counselling, Flipbooks<br>2)Follow-up visits	24 months	1) SBP<br>2) FBG<br>3)Change in self-reported mean number of daily cigarettes/bidis smoked from visit 1 to post-intervention	No significant difference in FBG between intervention and control group (p= 0.029). However in unadjusted analysis, the decrease in FBG was greater (difference of -26.6 mg/dl; 95% CI: -2.7 to -50.7mg/dl) in the intervention group (-43.0 ±83.5 mg/dl) than in the control group (-16.3 ± 77.2mg/dl). Significant reduction in SBP between intervention and control group.
Schwalm, 2019	1371 Patients with hypertension	Cluster RCT	Non Physician Health Workers (NPHW)	1)Non-pharmacologic- Community screening, detection and treatment by tablet based management algorithms and counselling<br><br>Pharmacological- Free antihypertensive medications and a statin recommended by NPHWs, supervised by local physicians	12 months	1)Framingham Risk Score<br>2)SBP<br>3) LDL, HDL, total cholesterol, Triglycerides<br>4)Glucose concentrations	Significant difference of change in FRS 10year cardiovascular disease risk of –4·78% (95% CI –7·11 to –2·44; p<0·000) between control and intervention group. However, there were no significant differences in glucose concentrations.
Azami, 2018 (Iran)	142, Patients with diabetes mellitus	RCT	Nurse	1)Non pharmacologic - group sessions<br>2)Telephone follow-up	12 weeks	1)HbA1c<br>2) Lipid profile<br>3) BP<br>4)Body weight	Significant improvement of HbA1c, blood pressure, body weight, efficacy expectation, outcome expectation, and diabetes self-management behaviours in the intervention group.
Nesari, 2010 (Iran)	60, Patients with diabetes mellitus	RCT	Nurse	1)Telephonic follow-up<br>2)Pharmacologic- Dose adjustment	12weeks	1)HbA1c	Significant improvement in HbA1c levels for experimental group after 12 weeks intervention.(-1.87%, *P* □□0.001 for the experimental group *vs* -0.4%, *P* □□0.15 for the control group)

Farsaei, 2011 (Iran)	174, Patients with diabetes mellitus	RCT	Pharmacist	1)Non pharmacologic-education<br>2)Telephonic follow-up	3 Months	1)HbA1c<br>2)Fasting blood glucose	Significant reduction in HbA1c and FBG in the intervention group at the end of the study period.
Jarab, 2012 (Jordan)	156, Patients with diabetes mellitus	RCT	Pharmacist	1)Non pharmacologic-education, counselling<br>2)Telephonic follow-up<br>3)Pharmacologic- Dose adjustment	6 Months	1)Reduction in HbA1c<br>2) BP<br>3)Cholesterol<br>4)Triglycerides<br>5)BMI	Intervention patients who received clinical pharmacy services showed a mean reduction in A1c of 0.8% over 6 months, while the usual care group had a mean increase of 0.1% in A1c compared with baseline. Compared with baseline values, the intervention patients showed a mean reduction of 2.3 mmol/L, while usual care patients had a mean increase of 0.9 mmol/L in fasting blood glucose (FBG) at the 6-month assessment.<br>Significant reduction of HbA1c (-0.8% [-1.6 to 0.1] vs. +0.1 [-0.4 to 0.7]); , fasting blood glucose, systolic BP, diastolic Bp, total cholesterol, serum triglycerides for pharmaceutical care patients than usual care patients after 6 month follow-up.
Zhang, 2017 (China)	199, Patients admitted with a medical diagnosis of angina or myocardial infarction	RCT	Nurse	1) Non pharmacologic-education, counselling and problem assessment	7 months	1) Clinical outcomes	Significant differences in majority of clinical outcomes between the control and intervention groups over time which included fasting blood glucose (FPG, t= 2.249, P = 0.027), systolic blood pressure (SBP, t= 5.762, P = 0.000), diastolic blood pressure (DBP, t = 4.250, P =0.000).
Hammad, 2011 (Jordan)	199, Patients with metabolic syndrome	RCT	Pharmacist	1)Non pharmacologic-education, counselling, evaluating metabolic syndrome components, prepare patient care plan, monitoring<br>2)Pharmacologic-initiation of drug	6 months	1)Waist circumference<br>2)Elevated triglycerides<br>3) Low HDL-C<br>4)Elevated BP	No significant differences between the intervention and control arms for FBG.

				therapy based on clinical guidelines and in consultation with the physician		5)Elevated fasting blood glucose<br>6)Body weight	
Plaster, 2012 (Brazil)	63, Type 2 diabetes patients with metabolic syndrome	RCT	Pharmacist	1) Non pharmacologic-Education, interviewing<br><br>2)Biochemical and anthropometric measurements	6 months	1)Anthropometric and biochemical parameters	Significant reduction in Plasma glucose (mg/dL$^{-1)}$ for final intervention with final control group (173±13 Vs 130±12; P<0.01).Nearly all clinical parameters evaluated were improved at the end of the study in the Intervention group when compared with their initial values.
Saffi, 2014 (Brazil)	74, Patients with Coronary Artery Disease (CAD) diagnosis by catheterization, treatment of acute coronary syndrome	RCT	Nurse	1)Non pharmacologic-counselling<br>2)Telephonic counselling	12 months	1) Lipid profile<br>2) Blood glucose<br>3) HbA1c<br>4) Weight<br>5) BMI,<br>6) WC, WHR<br>7) BP<br>8) Capillary blood glucose measurements	No statistical difference in laboratory variables at the end of one year between groups, before or after adjustment for covariance, except for change in LDL-C.
Muchiri, 2016 (South Africa)	82, Patients with diabetes mellitus	RCT	Dietician	1) Non pharmacologic-education	12 months	1)Changes in HbA1c in 6 months<br>2)Changes in BMI<br>3)Blood pressure and blood lipids<br>4)HbA1c and dietary behaviours at 12 months.	Post-intervention, mean difference between the intervention and control groups for HbA1c was −0·64% (95 % CI −0·19, 1·50 %; P=0·13) at 6 months<br>and −0·63% (95% CI 0·26, 1·50 %; P=0·16) at 12 months.<br><br>No significant improvement in mean difference between intervention and control groups for Hb A1c [−0·64% (95 % CI −0·19, 1·50 %; P=0·13) at 6 months and −0·63% (95% CI 0·26, 1·50 %; P=0·16) at 12 months] after the intervention.

Yin, 2018 (China)	184, Females with pre-diabetes, overweight or obese, not physically active and expressed interest on lifestyle changes.	RCT	Community health educator	2) Non pharmacologic-education, counselling	12 months	1)Weight<br>2) Waist circumference<br>3)HbA1c<br>4) FPG<br>5)Heart rate	No statistically significant reduction in HbA1c has been reported. However, 18.9% and 26.2% of all participants lowered their HbA1c more than 10% at 6 months and 12 months follow up.
Huang, 2017 (China)	102, Patients with CHD event or death risk more than 20%.	RCT	Nurse	1)Non pharmacologic-education,<br>2)Follow up<br>3)Home visits	6 months	1)Systolic Blood Pressure<br>2) Glucose<br>3)Cholesterol<br>3)Body Mass Index (BMI)	Significant reduction in glucose levels (t =-2.49, p=0.015), cholesterol (t = -2.44, P= .017), and BMI (t= -2.58, p= .011).) for participants in the intervention group compared to the usual care group.
Goldhaber-Fiebert, 2003 (Costa Rica)	75, Patients with diabetes mellitus	RCT	Nutritionist	1) Non pharmacologic-education	12 weeks	1)Weight<br>2) Blood pressure<br>3)Glycosylated haemoglobin<br>4) Fasting plasma glucose<br>5)Serum total cholesterol<br>6)HDL<br>7)Triglyceride levels and cholesterol	Significant reduction in Fasting plasma glucose for intervention group (19 ± 55 mg/dl) and increase (16 ± 78 mg/dl) in the control group (*P* =0.048). Glycosylated haemoglobin decreased 1.8 ±2.3% in the intervention group and 0.4 ±2.3% in the control group (*P* = 0.028).
Chao, 2012 (China)	1962, General population	RCT	Community health service centre staffs, managers	1)Non pharmacologic-education, counselling<br>2) Skill training	18 months	1)BMI<br>2)Waist to hip ratio<br>3)Blood Pressure	Significant improvements in the management group were observed in fasting blood sugar compared with the control group.

				3) Telephonic consultation		4)Triglycerides<br>5)Fasting Plasma Glucose	
Wishah,2015<br><br>(Jordan)	106, Patients with diabetes mellitus	RCT	Pharmacist	1)Pharmacologic- Dose adjustment<br>2)Non-pharmacologic- Education, counselling,<br>3)Telephonic follow up	6 months	1)HbA1c<br>2)FBS<br>3)LDL,HDL<br>4)Triglycerides<br>5)Total cholesterol<br>6)Weight<br>7)BMI<br>8)Blood Pressure	Significant reduction in mean of HbA1c and FBS of the patients in the intervention group compared to control group after 6 month follow-up (P<0.05).
Samtia, 2013 (Pakistan)	348, Patients with diabetes mellitus and were having BMI more than 25	RCT	Pharmacist	1)Non pharmacologic- education, counselling,<br>2)Smoking cessation	5 months	1)BMI<br>2)Waist circumference<br>3)FBS<br>4) HbA1c	Significant reduction in fasting blood glucose and HbA1c values in the intervention group . Reductions were seen in fasting blood glucose and HbA1c values in control group but were insignificant. Mean differences of fasting blood glucose and HbA1c values between control and intervention group from baseline were not significant.
Jahangard-Rafsanjani, 2015 (Iran)	95, Patients with diabetes mellitus	RCT	Community pharmacist	1)Non pharmacologic- Education<br>2)Telephone follow up,<br>3)Training on self-monitoring device	5 months	1)HbA1c<br>2) Blood Pressure<br>3) Weight<br>4) BMI	However, No significant difference was observed between study groups at the end of trial period.<br>At the end of trial, patients in intervention group achieved A1C level of 5.8+/- 0.8 while A1c increased in the control group upto 6.7+/- 1.4 and the difference between groups were significant (p=0 .02)
Chung, 2014 (Malaysia)	241, Patients with diabetes mellitus	RCT	Pharmacist	1)Non pharmacologic- Medication review, education, counselling<br>2) Training on self-monitoring<br>3)Telephonic follow up,	12 months	1)HbA1c<br>2) FBG	Significant differences in FBG and HbA1c were observed between the control and intervention groups at 4 months after intervention, and these continued until the end of the study period.

Sriram, 2011 (India)	120, Women with diabetes mellitus	RCT	Pharmacist	1)Non pharmacologic - education, counselling<br>2)Instructions on lifestyle and dietary regulations regarding their prescribed drugs	8 months	1)FBS<br>2)HbA1c<br>3) BMI	Significant reduction in average Hb A1c values from 8.44 ± 0.29% to 6.73 ± 0.21% ($p < 0.01$). There was a significant decrease in the fasting blood glucose from 195.57 ± 10.10 mg/dl to 107.25 ± 3.70 mg/dl between the baseline and the final interview in the intervention group ($p < 0.01$).
Hailu, 2018 (Ethiopia)	142, Patients with diabetes mellitus either overweight or obese	RCT	Nurse	1)Non pharmacologic- Facilitating DSME sessions<br>2)Telephonic follow-up	9 months	1)HbA1c<br>2)Mean differences in HbA1c<br>3) FBS<br>4)Blood Pressure<br>5) BMI<br>6) Waist circumference	Significant reduction in mean HbA1c between both intervention and comparison groups. But end line mean HbA1c difference between groups were not significant. Significantly lower FBS at end line in the intervention group as adjusted for sociodemographic and clinical factors.
Sun, 2008 (China)	150,Patients with diabetes mellitus and BMI > 23kg/m2	RCT	Nutritionist	1)Non pharmacologic- education, consultations	24 weeks	1)Body weight<br>2)Blood Pressure<br>3)Waist and hip circumference<br>4)FPG<br>5)insulin<br>6)Lipids<br>7)HbA1c	Significant improvement in fasting blood glucose, insulin, systolic and diastolic blood pressures for intervention group compared to reference Group ($p<0.05$). Importantly, HbA1c was lower ($p<0.001$) in the Intervention Group at 12 weeks (-0.6 ± 0.1%) and 24 weeks (-0.8 ± 0.1%). Weight loss was modest, but significant differences were observed between groups ($p<0.05$).
Scain, 2009 (Brazil)	104, Patients with diabetes mellitus	RCT	Nurse educator	1)Non pharmacologic- education	1 year	1)HbA1c<br>2)Weight<br>3)Blood Pressure<br>4) Lipids	.<br><br>Significant reduction in HbA1c levels after the 4th month and remained lower than in the control group until the 12th month. Despite remaining significant, the difference

							diminished over the subsequent months, indicating that the effect of education on A1C loses some impact over time.
Liang, 2012 (China)	59, Patients with diabetes mellitus with high risk of developing diabetic ulcers	RCT	Diabetes nurse led multidisciplinary team including 3 endocrinologists, 4 nurses, 1 dietician	1)Non pharmacologic-education, demonstration, clinical follow up<br>2)Telephonic reminder	2 years	1)HbA1c	Significant difference between groups in HbA1c values at the 2 year follow up.
Cani, 2015 (Brazil)	70, Patients with diabetes mellitus	RCT	Pharmacist	1)Non pharmacologic-education<br>2)Pharmacologic- Drug adjustments	6 months	1)Change in HbA1c levels	Significant improvement in the mean HbA1c levels in intervention group 9.21(1.41) compared to control group 9.53(1.68). The baseline mean serum HbA1c level was 9.61(1.38) in the CG and 9.78(1.55) in the IG therefore corresponding to a decrease of 0.57%
Mahwi, 2013 (Iraq)	123, Patients with diabetes mellitus	RCT	Pharmacist	1)Non pharmacologic-education sessions<br>2)Use of diary logs<br>3)Follow up calls and appointments	4 months	1)FPG<br>2) HbA1c	Significant reduction in the HbA1c levels and FBG (9.19 ± 2.0% and 197.5 ± 77.1mg/dl respectively) of patients in the intervention group at the end of the study. But there is no significant glycemic reduction in the control group.
De Souza, 2017 (Brazil)	118, Patients with diabetes mellitus	RCT	CHW	1)Non pharmacologic-education<br>2) Home visits	3 months	1)HbA1c<br>2) BMI<br>3) Blood Pressure<br>4) FPG<br>5)HDL<br>6)Creatinine, TG<br>7)Albuminuria	Significant reduction in HbA1c levels was observed in both the groups overtime. ut no between group differences were observed. No details on FPG.

Mollaoğlu ,2009 (Turkey)	50, Patients with diabetes mellitus	RCT	Nurse	1)Non pharmacologic-education<br>2) Home visits	3 months	1)HbA1c<br>2)Blood glucose<br>3)Urine glucose value<br>4)Cholesterol level (total cholesterol, triglycerides, HDL and LDL)	Significant differences were found between fasting blood sugar, postprandial blood sugar, urine glucose, haemoglobin A1c (9.5 ± 1.7 mg/dl to 7.5 ± 1.3 mg/dl), triglyceride, total cholesterol, and low density lipoprotein cholesterol levels after the education program. The FBS values of the experimental group individuals fell from high to acceptable values but the control group individuals' FBS values remained high. Difference between PPBS values the two groups was found to be statistically significant at the final measurement
Jayasuriya, 2013/2015 (Sri Lanka)	53, Patients with diabetes mellitus diagnosed within the last 5 years	RCT	Nurse	1) Non pharmacologic-education, motivational interviewing	6 months	1)1% achieving HbA1c target 6.5%, diet<br>2)Physical activity<br>3)Blood Pressure<br>4) Lipids<br>5) Waist circumference waist hip ratio<br>6)Body weight	and other measurements of variables. Significant improvement in the mean HbA1c of the intervention group 9.8% to 7.0% ($P < 0.001$) and from 9.7% to 8.3% ($P < 0.005$) in the "usual care" group at 6 months . A significant difference between the groups was found in HbA1c ($P = 0.035$) after controlling for baseline differences and other measurements of variables.
Oberli-Neto, 2011 (Brazil)	194, Patients with diabetes and/or hypertension	RCT	Pharmacist	1)Non pharmacologic-Problem assessment, suggestion to physician regarding new drug regimen, developing individualised patient care plans, education and counselling group sessions<br>2) Follow up evaluation.<br>3)Pharmacologic- Dose	36 months	1)Blood Pressure<br>2)Weight,<br>3)Abdominal circumference<br>4)Lipids<br>5) HbA1c	Significant reductions in the mean values (baseline vs. 36 months; 95% confidence interval [CI]) of fasting glucose and hemoglobin A1C were observed in the intervention group, but no significant changes were found in the control group. The difference between the groups in the change observed over 36-month follow-up was significant ($P < 0.05$).

				adjustment, modification of drug therapy (addition or withdrawal)			
Wattana ,2007 (Thailand)	147, Patients with diabetes mellitus	RCT	Nurse	1)Non pharmacologic - education<br>2) Home visits	24 weeks	1)HbA1c	Significant reduction in HbA1c for experimental group compared to control group at 24 weeks. ($F(1,143) = 6.19$, $P < 0.05$). The mean scores of the HbA_{1c} of the experimental group decreased from 8.08% at baseline to 7.40% at 24 weeks, whereas the control group decreased from 8.09% to 8.02%
Phumipamorn, 2008 (Thailand)	130, Patients with diabetes mellitus	RCT	Pharmacist	1) Nonpharmacologic- Pill count, refill medication, education	10 months	1)Change in the HbA1c level<br>2)Changes in lipid profiles	No significant reduction in HbA1c levels between study group and control group (−0.8 vs. −0.6, $p = 0.56$). A significant fall in the total cholesterol was achieved in the study group (−31.6 mg/dL, 95% CI [−41.9, −21.3]) when compared to the control group (−1.2 mg/dL, 95% CI [−14.0, 11.7]; $P = 0.000$).
Lim, 2016 (Malaysia)	76, Patients with diabetes mellitus	RCT	Pharmacist	1)Nonpharmacologic- education, counselling<br>2)Blood glucose monitoring<br>3)Pharmacologic- Dose adjustments/addition, ordering indicated lab tests after discussion and approval by the doctor	16 months	1)HbA1c<br>2) FBG<br>3) BMI<br>4) Blood Pressure<br>5)LDL, HDL, total cholesterol, triglyceride	No significant difference of baseline HbA1c and FBG between intervention and control groups. The mean HbA1c in the intervention group decreased significantly from 10.11% to 9.21% (p=0.001). However, the reduction of mean HbA1c in the non-intervention group was not significant (p=0.491). In addition, the FBG was significantly improved in the intervention group with a reduction of 3.45mmol/l (p=0.002) and there was significant difference of mean FBG between the intervention and non-intervention groups.

Mourao, 2012/2013 (Brazil)	100, Patients with diabetes mellitus	RCT	Pharmacist	1) Non pharmacologic- Needs assessment, designing care plan, education	6 months	1)HbA1c 2)Fasting plasma glucose 3)Total cholesterol, LDL cholesterol, HDL cholesterol, triglycerides 4)Blood Pressure	. Significant improvement of HbA1c (-0.6 vs 0.7 %, p = 0.001), fasting plasma glucose, total cholesterol, LDL cholesterol, triglycerides and systolic blood pressure and a significant increase in HDL cholesterol and the use of lipid-modifying agents and platelet aggregation inhibitors. No significant difference in BMI or diastolic blood pressure was found
Ali, 2016 (India and Pakistan)	1146, Patients with diabetes mellitus	RCT	Non physician clinical coordinator	1)Non pharmacologic - Entry into DS HER 2) Telephonic follow-up	36 months	1)Proportion of patients from each group achieving HbA1c level less than 7% plus a BP less than 130/80 mm Hg and/or an LDLc level less than 2.59 mmol/L (<100 mg/dL) (<1.81 mmol/L [<70 mg/dL] for patients with a history of cardiovascular disease [CVD])	Intervention participants achieved greater reductions in HbA1c level (_0.50% [CI, _0.69% to _0.32%]), systolic BP (_4.04 mm Hg [CI, _5.85 to _2.22 mm Hg]), diastolic BP (_2.03 mm Hg [CI, _3.00 to _1.05 mm Hg]). No relative or absolute differences in the between group likelihood of achieving the primary outcome
Moreira, 2015 (Brazil)	77, Patients with diabetes mellitus	RCT	Nurse	1)Non pharmacologic- education 2)Telephonic follow up and remainder	12 months	1)HbA1c	Significant reduction in HbA1c levels in both the intervention (p < .01) and control (p =.05) groups over time. However, the absolute reductions in HbA1c of −0.41 between 6 months and baseline (p = .27) and of 0.69

							between 6 and 12 months (p = .08) were not significant
Shao, 2017 (China)	199, Patients with diabetes mellitus	RCT	Pharmacist	1)Non-pharmacological intervention- health education, face to face and telephone interview	6 months	1)Fasting blood glucose (FBG)<br>2) HbA1c	Significant improvement in FBG (P<0.05) and HbA1c (P<0.05) after 6-month intervention in intervention group, while no significant changes were observed in control group. The ratio of patients who reached the target HbA1c level in the IG increased to 76.0%, which was significantly higher than that of CG (47.5%, P<0.05) and that of IG before intervention (57.0%, P<0.05)
Sartorelli et al, 2005 (Brazil)	104, High-risk group (overweight and relatives of DM patients)	RCT	Nutritionist	1)Non-pharmacologic-Individualised dietary counselling session, health checks, diet prescription with food exchange list	12 months	1)Body composition<br>2) Biochemical indicators and lifestyle	Significant reduction in body weight, waist circumference, diastolic blood pressure, fasting blood glucose, and cholesterol levels in intervention group than control group after 6 month follow-up(P<0.05). At 6- and 12-month follow-up, the proportion of subjects in the intervention group who increased their intake of fruits ,use of olive oil and reduced their intake of saturated fat was significantly higher than in the control group.
Guo 2019 (China)	171,Patients with diabetes mellitus	RCT	Nurse	1)Non-pharmacologic-follow-up visits, health lectures, and free diabetes expert consultation services.	12 months	1)FBG<br>2)HbA1c<br>3) Diabetes self-management	Significant difference in HbA1c between the two groups (F=10.114, *P*<0.05) after 12 months of intervention. But the intervention had no significant effect on fasting blood sugar.
Ma 2014 (China)	120, Patients with hypertension	RCT	Nurse	1)Non-pharmacologic-adapted Motivational Interviewing (MI), recording daily diary on medication adherence and lifestyle	24 weeks	1)Blood pressure<br>2)Lab test values (triglycerides, total cholesterol)<br>3) Fasting blood glucose	No significant difference in all the lab values (SBP, DBP, FBG, postprandial blood sugar, serum creatinine, total cholesterol, triglycerides, LDL, HDL) between intervention and control group and within the subjects in the groups. However, SBP and DBP of the

						4) Treatment adherence<br>5)General self-efficacy	intervention group decreased compared with ones of the control group over MI counselling, the difference values were 4.92 and 2.58, respectively, which had a statistical difference between two groups

RCT= Randomised controlled trial, cRCT=Cluster randomised trial, DM=Diabetes Mellitus, HbA1c=glycated haemoglobin, FBG=Fasting Blood Glucose, BMI=Body Mass Index, CHW=Community Health Worker, CVD=Cardiovascular Disease, PCP=Primary care physician, CAD= Coronary Artery Disease, SD=standard deviation

Table S2: PRISMA checklist

Section/topic	#	Checklist item	Reported on page #
**TITLE**			
Title	1	Identify the report as a systematic review, meta-analysis, or both.	1
**ABSTRACT**			
Structured summary	2	Provide a structured summary including, as applicable: background; objectives; data sources; study eligibility criteria, participants, and interventions; study appraisal and synthesis methods; results; limitations; conclusions and implications of key findings; systematic review registration number.	1
**INTRODUCTION**			
Rationale	3	Describe the rationale for the review in the context of what is already known.	2
Objectives	4	Provide an explicit statement of questions being addressed with reference to participants, interventions, comparisons, outcomes, and study design (PICOS).	2,3
**METHODS**			
Protocol and registration	5	Indicate if a review protocol exists, if and where it can be accessed (e.g., Web address), and, if available, provide registration information including registration number.	2
Eligibility criteria	6	Specify study characteristics (e.g., PICOS, length of follow-up) and report characteristics (e.g., years considered, language, publication status) used as criteria for eligibility, giving rationale.	2
Information sources	7	Describe all information sources (e.g., databases with dates of coverage, contact with study authors to identify additional studies) in the search and date last searched.	3
Search	8	Present full electronic search strategy for at least one database, including any limits used, such that it could be repeated.	Online supplemen t
Study selection	9	State the process for selecting studies (i.e., screening, eligibility, included in systematic review, and, if applicable, included in the meta-analysis).	3
Data collection process	10	Describe method of data extraction from reports (e.g., piloted forms, independently, in duplicate) and any processes for obtaining and confirming data from investigators.	3
Data items	11	List and define all variables for which data were sought (e.g., PICOS, funding sources) and any assumptions and simplifications made.	3

Risk of bias in individual studies	12	Describe methods used for assessing risk of bias of individual studies (including specification of whether this was done at the study or outcome level), and how this information is to be used in any data synthesis.	Online supplement
Summary measures	13	State the principal summary measures (e.g., risk ratio, difference in means).	4
Synthesis of results	14	Describe the methods of handling data and combining results of studies, if done, including measures of consistency (e.g., I^2) for each meta-analysis.	4

Table S3: Cochrane risk of bias

**Author, year**	**Type**	**Random sequence generation (Selection bias)**	**Allocation concealment (Selection bias)**	**Blinding of participants and personnel (Performance bias)**	**Blinding of outcome assessment (Detection bias)**	**Incomplete outcome data (Attrition bias)**	**Selective outcome reporting (Reporting bias)**
Huang 2017	RCT	-	?	?	?	-	+
Chung 2014	RCT	-	?	-	?	-	+
Liang 2012	RCT	-	?	-	-	-	
Phumipamorn 2008	RCT	-	?	+	+	?	+
Lim 2016	RCT	-	-	?	+	-	+
Labhardt 2011	cRCT	?	+	-	?	-	-
Khetan 2019	cRCT	?	?	+	+	-	+
Farsaei 2011	RCT	?	?	+	+	-	+
Samtia 2013	RCT	?	?	?	?	?	-
Sun 2008	RCT	?	?	-	?	?	+
Cani 2015	RCT	?	-	?	?	+	+
Mahwi 2013	RCT	?	?	?	?	+	+
Mollaoğlu 2009	RCT	?	?	?	?	+	+
Wattana 2007	RCT	?	?	?	?	?	-
Sartorelli 2007	RCT	?	?	-	?	-	?
Mash 2014	cRCT	+	+	-	+	-	+
Fairall 2016	cRCT	+	?	+	?	?	?
Depue 2013	cRCT	+	?	-	+	+	+
Ramli 2016	cRCT	+	?	?	?	+	+
Prabhakaran 2018	cRCT	+	+	-	+	+	+
Schwalm 2019	cRCT	+	+	+	+	+	+
Azami 2018	RCT	+	+	-	+	+	+
Nesari 2010	RCT	+	?	?	+	+	+
Jarab 2012	RCT	+	?	?	?	+	+
Zhang 2017	RCT	+	+	+	+	+	+
Hammad 2011	RCT	+	?	-	+	?	+
Plaster 2012	RCT	+	?	+	+	?	+
Saffi 2014	RCT	+	+	-	+	-	+
Muchiri 2016	RCT	+	+	+	+	+	+
Yin 2018	RCT	+	?	?	?	+	+
Goldhaber-Fiebert 2003	RCT	+	?	-	?	?	-
Wishah 2015	RCT	+	?	-	?	+	+

Jahangard 2015	RCT	+	+	?	?	-	+
Sriram 2011	RCT	+	?	?	?	?	+
Scain 2009	RCT	+	?	-	?	+	+
De Souza 2017	RCT	+	+	-	+	+	?
Hailu 2018	RCT	+	?	-	+	+	+
Jayasuriya 2015	RCT	+	+	+	?	-	-
Obreli –Nato 2011	RCT	+	?	?	?	?	-
Mourao 2013	RCT	+	?	?	+	+	+
Ali 2016	RCT	+	+	+	?	+	+
Moreira 2015	RCT	+	+	+	+	+	+
Guo 2019	RCT	+	-	+	-	-	-
Ma 2014	RCT	+	?	-	-	+	+
Chao 2012	RCT	+	+	-	-	+	+
Shao 2017	RCT	-	?	-	-	+	+

?	Unclear risk
-	High risk
+	Low risk

Table S4: HbA1c meta-analysis heterogeneity assessment, by omitting single study and estimating the change in overall heterogeneity (I^2)

Author, year	***Effect***	***LLCI***	***ULCI***	***I^2***
Omitting Yin 2018	*-0.606*	*-0.817*	*-0.395*	*0.797*
Omitting Sriram 2011	*-0.548*	*-0.798*	*-0.297*	*0.905*
Omitting Azami 2018	*-0.534*	*-0.809*	*-0.260*	*0.949*
Omitting Obreli 2011	*-0.575*	*-0.867*	*-0.283*	*0.950*
Omitting Wishah 2015	*-0.540*	*-0.820*	*-0.260*	*0.953*
Omitting Scain 2009	*-0.585*	*-0.871*	*-0.299*	*0.955*
Omitting Goldhaber-Fiebert 2003	*-0.554*	*-0.836*	*-0.273*	*0.955*
Omitting Ali 2016	*-0.591*	*-0.883*	*-0.300*	*0.955*
Omitting Nesari 2010	*-0.546*	*-0.827*	*-0.265*	*0.955*
Omitting Jayasuriya 2015	*-0.553*	*-0.834*	*-0.272*	*0.955*
Omitting Guo 2019	*-0.573*	*-0.859*	*-0.288*	*0.955*
Omitting Depue 2013	*-0.594*	*-0.881*	*-0.308*	*0.956*
Omitting Moreira 2015	*-0.580*	*-0.863*	*-0.297*	*0.956*
Omitting Mourao 2013	*-0.565*	*-0.846*	*-0.285*	*0.956*
Omitting Saffi 2014	*-0.608*	*-0.892*	*-0.323*	*0.956*
Omitting De Souza 2017	*-0.584*	*-0.868*	*-0.300*	*0.956*
Omitting Hailu 2018	*-0.586*	*-0.868*	*-0.304*	*0.956*
Omitting Prabhakaran 2018	*-0.607*	*-0.897*	*-0.316*	*0.956*
Omitting Jahangard 2015	*-0.584*	*-0.869*	*-0.300*	*0.956*
Omitting Jarab 2012	*-0.571*	*-0.853*	*-0.289*	*0.956*
Omitting Ramli 2016	*-0.602*	*-0.893*	*-0.311*	*0.956*
Omitting Fairall 2016	*-0.620*	*-0.908*	*-0.332*	*0.956*
Omitting Mash 2014	*-0.608*	*-0.895*	*-0.322*	*0.956*
Omitting Muchiri 2016	*-0.602*	*-0.886*	*-0.319*	*0.956*

Table S4.1 Univariate meta-regression for HbA1c meta-analysis

Covariates	Regression coefficient	95% CI	p_value	R^2
**Task shift group**				
CHW	*Reference*			
Dietitian	*-0.414*	*-1.16;0.33*	*0.277*	*64.66%*
Nurse	*-0.440*	*-1.00;0.11*	*0.123*	
Pharmacist	*-0.847*	*-1.46;-0.22*	*0.007*	
**WHO region**				
AFRO	*Reference*			
EMRO	*-1.232*	*-1.85;-0.61*	*<0.001*	*71.50%*
PAHO	*-0.648*	*-1.24;-0.04*	*0.034*	
SEARO	*-0.657*	*-1.26;-0.05*	*0.033*	
WPRO	*-0.282*	*-0.88;0.31*	*0.354*	
**Study population**				
Other conditions	*Reference*			
T2DM	*-0.559*	*-1.09;-0.02*	*0.041*	*46.04%*
**Duration of intervention month**	0.019	-0.01;0.05	0.246	0.00%
**Physician density**	-0.002	-0.03;0.03	0.887	9.75%
**Sample size**	0.001	-0.00;0.00	0.180	0.00%

In multivariate meta-regression we used a stepwise backward selection method for model building, and we eliminated non-significant predictors from the full model in a stepwise manner and selected the final model.

Table S4.2 Multivariate meta-regression for HbA1c (R^2 =93.4%)

Covariates	Regression coefficient	95% CI	p_value
**WHO region**			
AFRO	*Reference*		
EMRO	*-1.051*	*-1.54;-0.56*	*<0.001*
PAHO	*-0.372*	*-0.88;0.14*	*0.157*
SEARO	*-0.646*	*-1.10;-0.19*	*0.005*
WPRO	*-0.243*	*-0.65;0.17*	*0.250*
**Study population**			
Other conditions	*Reference*		
T2DM	*-0.502*	*-0.85;-0.15*	*0.004*
**Duration of intervention month**	-0.021	-0.04;-0.00	0.037
**Sample size**	0.001	0.00;0.00	<0.001

Table S5: FBS meta-analysis heterogeneity assessment, by omitting single study and estimating the change in overall heterogeneity (I^2)

Author, year	*Effect*	*LLCI*	*ULCI*	*I^2*
Omitting Yin 2018	*-18.610*	*-27.174*	*-10.047*	*0.825*
Omitting Obreli 2011	*-15.402*	*-22.663*	*-8.141*	*0.854*
Omitting Wishah 2015	*-14.346*	*-21.445*	*-7.247*	*0.865*
Omitting Sriram 2011	*-14.836*	*-22.115*	*-7.558*	*0.875*
Omitting Ma 2014	*-16.736*	*-24.596*	*-8.876*	*0.880*
Omitting Zhang 2017	*-16.384*	*-24.090*	*-8.678*	*0.882*
Omitting Prabhakaran 2018	*-19.175*	*-27.044*	*-11.305*	*0.883*
Omitting Plaster 2012	*-15.565*	*-22.963*	*-8.167*	*0.884*
Omitting Mourao 2013	*-15.882*	*-23.414*	*-8.350*	*0.885*
Omitting Chao 2012	*-17.090*	*-25.028*	*-9.152*	*0.885*
Omitting Goldhaber-Fiebert 2003	*-16.068*	*-23.604*	*-8.533*	*0.887*
Omitting Jarab 2012	*-16.313*	*-23.779*	*-8.848*	*0.888*
Omitting Saffi 2014	*-17.396*	*-25.147*	*-9.645*	*0.890*
Omitting Scain 2009	*-17.361*	*-25.134*	*-9.587*	*0.890*
Omitting Hailu 2018	*-16.980*	*-24.654*	*-9.306*	*0.890*
Omitting Hammad 2011	*-17.843*	*-25.772*	*-9.915*	*0.890*
Omitting Schwalm 2019	*-17.990*	*-26.143*	*-9.837*	*0.890*
Omitting Guo 2019	*-18.024*	*-26.097*	*-9.951*	*0.890*

<u>Table S5.1:</u> Univariate meta-regression for FBS meta-analysis

**Covariates**	**Regression coefficient**	**95% CI**	**p_value**	**R^2**
**Task shift group**				
CHW	*Reference*			
Dietitian	*-28.68*	*-69.34;11.96*	*0.166*	*26.10%*
Nurse	*-1.10*	*-18.17;15.96*	*0.898*	
Pharmacist	*-27.65*	*-45.92;-9.39*	*0.003*	
**WHO region**				
AFRO	*Reference*			
EMRO	*-14.98*	*-55.50;25.53*	*0.468*	*10.59%*
PAHO	*-12.28*	*-50.08;25.50*	*0.523*	
SEARO	*1.11*	*-39.91;42.13*	*0.957*	
WPRO	*2.19*	*-34.66;39.05*	*0.907*	
**Study population**				
Other conditions	*Reference*			
T2DM	*-9.86*	*-25.93;6.21*	*0.229*	*0.00%*
**Duration of intervention**				
**month**	0.232	-0.73;1.20	0.638	0.00%
**Physician density**	-0.81	-2.01;0.37	0.180	11.55%
**Sample size**	0.070	0.02;0.12	0.005	0.00%

Table S5.2 Multivariate meta regression by stepwise backward elimination (R^2 =36.75%)

Covariates	Regression coefficient	95% CI	p_value
**Task shift group**			
CHW	*Reference*		
Dietitian	*-18.95*	*-59.35;21.45*	*0.357*
Nurse	*1.49*	*-14.67;17.66*	*0.856*
Pharmacist	*-21.09*	*-39.17;-3.01*	*0.022*
**Sample size**	0.04	0.00;0.09	0.028

Table S6 GRADE Summary of findings

Summary of findings:

Task Sharing/Task shifting compared to Usual care for Type II Diabetes Management

Patient or population: Type II Diabetes Management

Setting: Low Middle Income Countries

Intervention: Task Sharing/Task shifting

Comparison: Usual care

Outcomes	Anticipated absolute effects* (95% CI)		Relative effect (95% CI)	№ of participants (studies)	Certainty of the evidence (GRADE)	Comments
	**Risk with Usual care**	**Risk with Task Sharing/Task shifting**				
HbA1c (%) change from the baseline (Task shifting with Nurses) follow up: median 12 months	The mean hbA1c (%) change from the baseline was **-0.54** %	MD **0.53 % lower** (0.89 lower to 0.18 lower)	-	2724 (12 RCTs)	⨁⨁⨁◯ MODERATE	
HbA1c (%) change from the baseline (Task shifting with CHW) follow up: median 12 months	The mean hbA1c (%) change from the baseline was **-0.52** %	MD **0.05 % higher** (0.03 higher to 0.06 higher)	-	557 (3 RCTs)	⨁⨁◯◯ LOW [a]	
HbA1c (%) change from the baseline (Task shifting with Dietitian) follow up: median 12 months	The mean hbA1c (%) change from the baseline was **-0.87** %	MD **0.5 % lower** (1.09 lower to 0.09 higher)	-	1303 (3 RCTs)	⨁⨁⨁◯ MODERATE	
HbA1c (%) change from the baseline (Task shifting with Pharmacist) follow up: median 6 months	The mean hbA1c (%) change from the baseline was **-0.12** %	MD **0.91 % lower** (1.14 lower to 0.68 lower)	-	761 (6 RCTs)	⨁⨁⨁◯ MODERATE	
Fasting Blood Sugar (mg/dl) change from baseline (Task shifting with	The mean fasting Blood Sugar (mg/dl) change from baseline was **-8.5** mg/dl	MD **7.46 mg/dl lower** (18.44 lower to 3.52 higher)	-	1428 (7 RCTs)	⨁⨁⨁◯ MODERATE [b,c]	

Nurses)<br>follow up: median 12 months						
Fasting Blood Sugar (mg/dl) change from baseline (Task shifting with CHW)<br>follow up: median 12 months	The mean fasting Blood Sugar (mg/dl) change from baseline was **-0.66** mg/dl	MD **5.41 mg/dl lower** (12.74 lower to 1.92 higher)	-	846<br>(3 RCTs)	⊕⊕◯◯<br>LOW [c,d]	
Fasting Blood Sugar (mg/dl) change from baseline (Task shifting with Dietitian)<br>follow up: median 3 months	The mean fasting Blood Sugar (mg/dl) change from baseline was **16** mg/dl	MD **35 mg/dl lower** (65.96 lower to 4.04 lower)	-	75<br>(1 RCT)	⊕◯◯◯<br>VERY LOW [a,e]	
Fasting Blood Sugar (mg/dl) change from baseline (Task shifting with Pharmacist)<br>follow up: median 6 months	The mean fasting Blood Sugar (mg/dl) change from baseline was **-3.62** mg/dl	MD **36.26 mg/dl lower** (52.6 lower to 19.92 lower)	-	938<br>(7 RCTs)	⊕⊕⊕◯<br>MODERATE [c,f]	

***The risk in the intervention group** (and its 95% confidence interval) is based on the assumed risk in the comparison group and the **relative effect** of the intervention (and its 95% CI).

CI: Confidence interval; **MD:** Mean difference

GRADE Working Group grades of evidence
High certainty: We are very confident that the true effect lies close to that of the estimate of the effect
Moderate certainty: We are moderately confident in the effect estimate: The true effect is likely to be close to the estimate of the effect, but there is a possibility that it is substantially different
Low certainty: Our confidence in the effect estimate is limited: The true effect may be substantially different from the estimate of the effect
Very low certainty: We have very little confidence in the effect estimate: The true effect is likely to be substantially different from the estimate of effect

Fig S1: HbA1c Funnel Plot

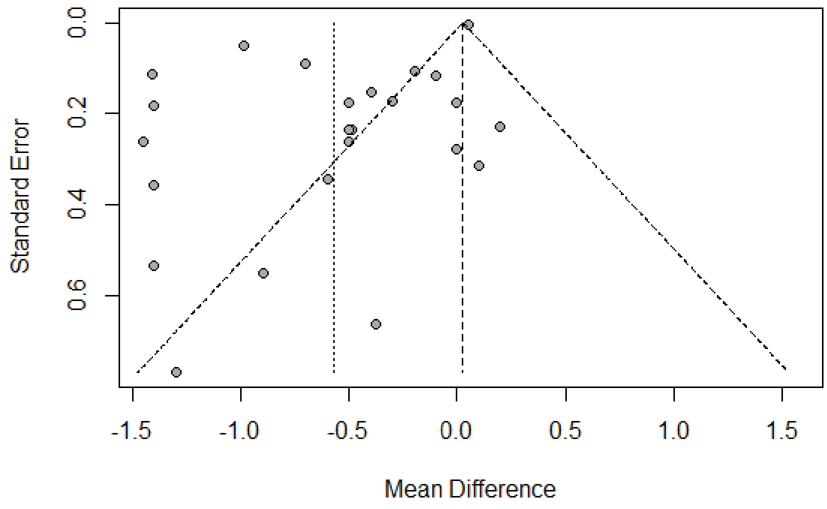

Fig S1.1: contour enhanced funnel plot for HbA1c meta-analysis

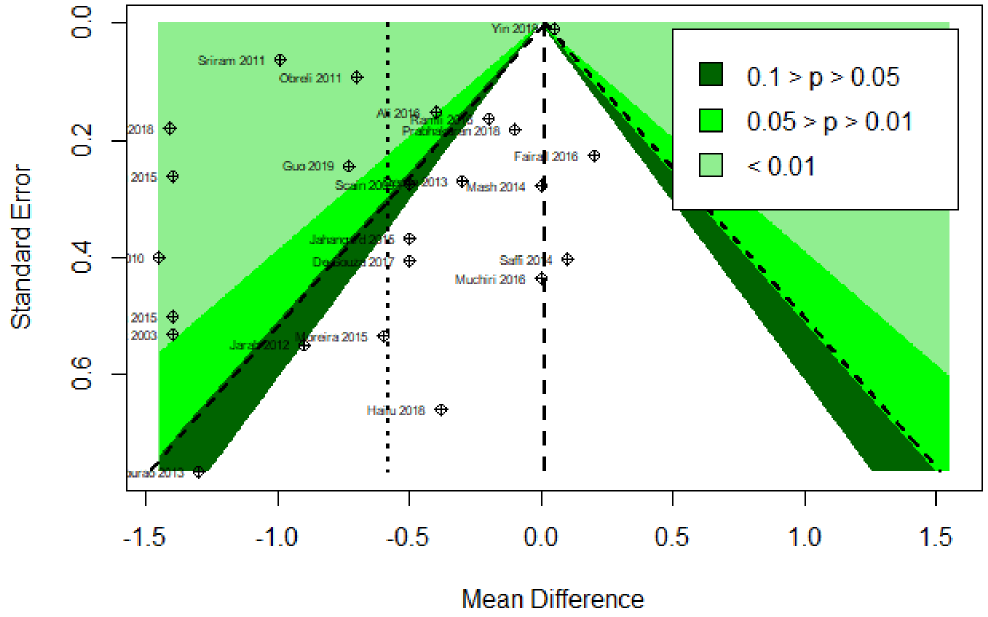

Fig S2: Sub-group analysis by task shared cadre (HbA1c) and type of intervention

Study	Experimental Total	Experimental Mean	Experimental SD	Control Total	Control Mean	Control SD	MD	95%-CI	Weight
**CHW: Non-Pharmacological Intervention**									
De Souza 2017	62	-1.20	2.07	56	-0.70	2.33	-0.50	[-1.30; 0.30]	3.8%
Yin 2018	109	-0.31	0.05	75	-0.36	0.06	0.05	[0.03; 0.07]	5.5%
Mash 2014	115	-0.50	2.17	140	-0.50	2.25	0.00	[-0.54; 0.54]	4.5%
Random effects model	286			271			0.05	[0.03; 0.07]	13.7%
Heterogeneity: $I^2 = 0\%$, $\tau^2 = 0$, $p < 0.01$									
**Dietitian: Non-Pharmacological Intervention**									
Goldhaber-Fiebert 2003	40	-1.80	2.30	35	-0.40	2.30	-1.40	[-2.44; -0.36]	3.1%
Ali 2016	575	-1.60	2.44	571	-1.20	2.68	-0.40	[-0.70; -0.10]	5.1%
Muchiri 2016	41	-1.00	1.86	41	-1.00	2.07	0.00	[-0.85; 0.85]	3.6%
Random effects model	656			647			-0.50	[-1.10; 0.09]	11.8%
Heterogeneity: $I^2 = 54\%$, $\tau^2 = 0.1520$, $p = 0.39$									
**Nurse: Non-Pharmacological Intervention**									
Depue 2013	104	-0.30	2.05	164	0.00	2.30	-0.30	[-0.83; 0.23]	4.6%
Moreira 2015	38	-1.30	2.26	39	-0.70	2.42	-0.60	[-1.65; 0.45]	3.1%
Saffi 2014	38	0.20	1.73	36	0.10	1.73	0.10	[-0.69; 0.89]	3.8%
Scain 2009	52	-0.30	1.41	52	0.20	1.40	-0.50	[-1.04; 0.04]	4.5%
Hailu 2018	78	-2.88	4.28	64	-2.50	3.59	-0.38	[-1.67; 0.91]	2.5%
Prabhakaran 2018	332	-1.90	2.25	328	-1.80	2.40	-0.10	[-0.46; 0.26]	5.0%
Azami 2018	71	-1.42	1.00	71	-0.01	1.13	-1.41	[-1.76; -1.06]	5.0%
Ramli 2016	314	-0.10	1.94	278	0.10	2.02	-0.20	[-0.52; 0.12]	5.1%
Jayasuriya 2015	28	-2.80	1.99	25	-1.40	1.65	-1.40	[-2.38; -0.42]	3.2%
Guo 2019	75	-0.58	1.49	68	0.15	1.42	-0.73	[-1.21; -0.25]	4.7%
Random effects model	1130			1125			-0.54	[-0.91; -0.18]	41.5%
Heterogeneity: $I^2 = 78\%$, $\tau^2 = 0.2386$, $p = 0.04$									
**Nurse: Pharmacological Intervention**									
Nesari 2010	30	-1.87	1.33	30	-0.42	1.74	-1.45	[-2.23; -0.67]	3.8%
Fairall 2016	180	0.00	2.40	229	-0.20	2.10	0.20	[-0.24; 0.64]	4.8%
Random effects model	210			259			-0.59	[-2.21; 1.02]	8.6%
Heterogeneity: $I^2 = 92\%$, $\tau^2 = 1.2556$, $p = 0.39$									
**Pharmacist: Non-Pharmacological Intervention**									
Mourao 2013	50	-0.60	3.80	50	0.70	3.87	-1.30	[-2.80; 0.20]	2.1%
Sriram 2011	60	-1.71	0.26	60	-0.72	0.40	-0.99	[-1.11; -0.87]	5.4%
Jahangard 2015	45	-1.00	1.55	40	-0.50	1.81	-0.50	[-1.22; 0.22]	4.0%
Random effects model	155			150			-0.98	[-1.10; -0.86]	11.5%
Heterogeneity: $I^2 = 0\%$, $\tau^2 = 0$, $p = 0.11$									
**Pharmacist: Pharmacological Intervention**									
Obreli 2011	97	-0.70	0.75	97	0.00	0.50	-0.70	[-0.88; -0.52]	5.3%
Jarab 2012	77	-0.80	4.00	79	0.10	2.72	-0.90	[-1.98; 0.18]	3.0%
Wishah 2015	52	-1.70	1.39	54	-0.30	1.30	-1.40	[-1.91; -0.89]	4.6%
Random effects model	226			230			-0.98	[-1.50; -0.45]	12.9%
Heterogeneity: $I^2 = 69\%$, $\tau^2 = 0.1389$, $p < 0.01$									
**Random effects model**	**2663**			**2682**			**-0.58**	**[-0.86; -0.30]**	**100.0%**
**Prediction interval**								**[-1.87; 0.71]**	

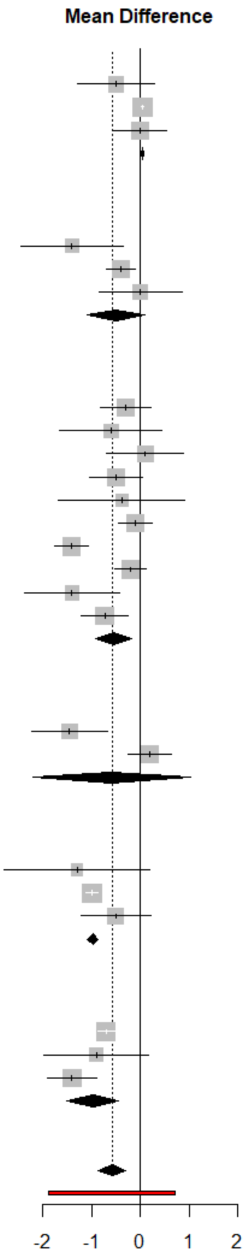

Heterogeneity: $I^2 = 95\%$, $\tau^2 = 0.3682$, $p < 0.01$
Residual heterogeneity: $I^2 = 74\%$, $p < 0.01$
Test for subgroup differences: $\chi^2_5 = 306.15$, df = 5 ($p < 0.01$)

Fig S3: Sub-group analysis by study population (HbA1c)

Study	Experimental Total	Experimental Mean	Experimental SD	Control Total	Control Mean	Control SD	MD	95%-CI	Weight
**Other conditions**									
Obreli 2011	97	-0.70	0.75	97	0.00	0.50	-0.70	[-0.88; -0.52]	5.3%
Saffi 2014	38	0.20	1.73	36	0.10	1.73	0.10	[-0.69; 0.89]	3.8%
Yin 2018	109	-0.31	0.05	75	-0.36	0.06	0.05	[0.03; 0.07]	5.5%
Fairall 2016	180	0.00	2.40	229	-0.20	2.10	0.20	[-0.24; 0.64]	4.8%
Random effects model	424			437			-0.12	[-0.63; 0.38]	19.4%
Heterogeneity: $I^2 = 96\%$, $\tau^2 = 0.2228$, $p < 0.01$									
**T2DM**									
Depue 2013	104	-0.30	2.05	164	0.00	2.30	-0.30	[-0.83; 0.23]	4.6%
Moreira 2015	38	-1.30	2.26	39	-0.70	2.42	-0.60	[-1.65; 0.45]	3.1%
Mourao 2013	50	-0.60	3.80	50	0.70	3.87	-1.30	[-2.80; 0.20]	2.1%
De Souza 2017	62	-1.20	2.07	56	-0.70	2.33	-0.50	[-1.30; 0.30]	3.8%
Scain 2009	52	-0.30	1.41	52	0.20	1.40	-0.50	[-1.04; 0.04]	4.5%
Goldhaber-Fiebert 2003	40	-1.80	2.30	35	-0.40	2.30	-1.40	[-2.44; -0.36]	3.1%
Hailu 2018	78	-2.88	4.28	64	-2.50	3.59	-0.38	[-1.67; 0.91]	2.5%
Prabhakaran 2018	332	-1.90	2.25	328	-1.80	2.40	-0.10	[-0.46; 0.26]	5.0%
Sriram 2011	60	-1.71	0.26	60	-0.72	0.40	-0.99	[-1.11; -0.87]	5.4%
Ali 2016	575	-1.60	2.44	571	-1.20	2.68	-0.40	[-0.70; -0.10]	5.1%
Azami 2018	71	-1.42	1.00	71	-0.01	1.13	-1.41	[-1.76; -1.06]	5.0%
Jahangard 2015	45	-1.00	1.55	40	-0.50	1.81	-0.50	[-1.22; 0.22]	4.0%
Nesari 2010	30	-1.87	1.33	30	-0.42	1.74	-1.45	[-2.23; -0.67]	3.8%
Jarab 2012	77	-0.80	4.00	79	0.10	2.72	-0.90	[-1.98; 0.18]	3.0%
Wishah 2015	52	-1.70	1.39	54	-0.30	1.30	-1.40	[-1.91; -0.89]	4.6%
Ramli 2016	314	-0.10	1.94	278	0.10	2.02	-0.20	[-0.52; 0.12]	5.1%
Mash 2014	115	-0.50	2.17	140	-0.50	2.25	0.00	[-0.54; 0.54]	4.5%
Muchiri 2016	41	-1.00	1.86	41	-1.00	2.07	0.00	[-0.85; 0.85]	3.6%
Jayasuriya 2015	28	-2.80	1.99	25	-1.40	1.65	-1.40	[-2.38; -0.42]	3.2%
Guo 2019	75	-0.58	1.49	68	0.15	1.42	-0.73	[-1.21; -0.25]	4.7%
Random effects model	2239			2245			-0.68	[-0.92; -0.44]	80.6%
Heterogeneity: $I^2 = 78\%$, $\tau^2 = 0.1777$, $p < 0.01$									
**Random effects model**	**2663**			**2682**			**-0.58**	**[-0.86; -0.30]**	**100.0%**
**Prediction interval**								**[-1.87; 0.71]**	

Heterogeneity: $I^2 = 95\%$, $\tau^2 = 0.3682$, $p < 0.01$
Residual heterogeneity: $I^2 = 86\%$, $p < 0.01$
Test for subgroup differences: $\chi^2_1 = 3.89$, df = 1 ($p = 0.05$)

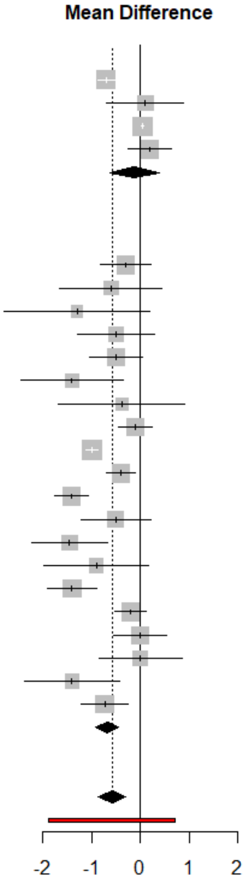

Fig S3.1: Sub-group analysis by sample size of each studies

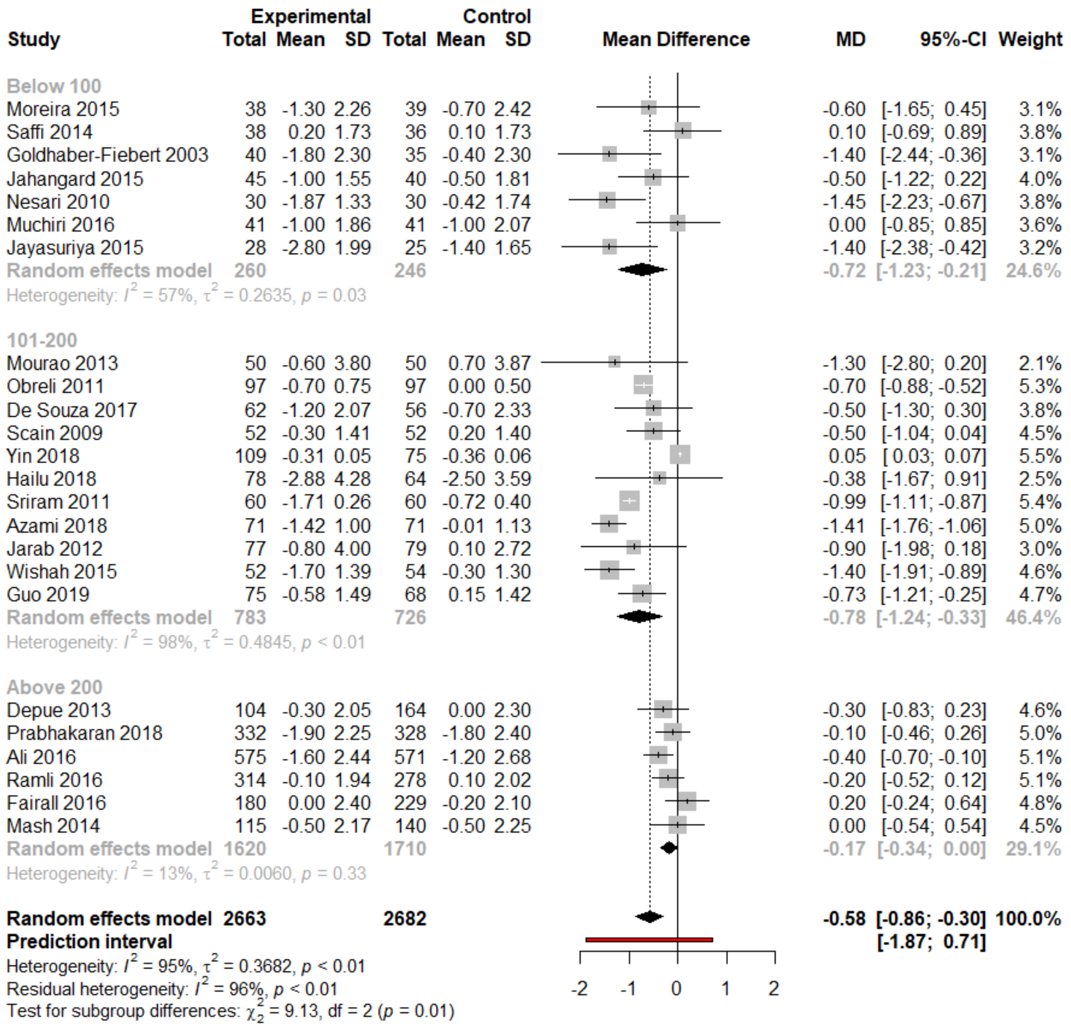

Fig S4: Sub-group analysis by follow-up (HbA1c)

Study	Experimental Total	Experimental Mean	Experimental SD	Control Total	Control Mean	Control SD	MD	95%-CI	Weight
**Upto 5 months**									
De Souza 2017	62	-1.20	2.07	56	-0.70	2.33	-0.50	[-1.30; 0.30]	3.8%
Goldhaber-Fiebert 2003	40	-1.80	2.30	35	-0.40	2.30	-1.40	[-2.44; -0.36]	3.1%
Azami 2018	71	-1.42	1.00	71	-0.01	1.13	-1.41	[-1.76; -1.06]	5.0%
Jahangard 2015	45	-1.00	1.55	40	-0.50	1.81	-0.50	[-1.22; 0.22]	4.0%
Nesari 2010	30	-1.87	1.33	30	-0.42	1.74	-1.45	[-2.23; -0.67]	3.8%
**Random effects model**	248			232			-1.08	[-1.53; -0.63]	19.6%
Heterogeneity: $I^2 = 53\%$, $\tau^2 = 0.1323$, $p = 0.08$									
**6-15 Months**									
Depue 2013	104	-0.30	2.05	164	0.00	2.30	-0.30	[-0.83; 0.23]	4.6%
Moreira 2015	38	-1.30	2.26	39	-0.70	2.42	-0.60	[-1.65; 0.45]	3.1%
Mourao 2013	50	-0.60	3.80	50	0.70	3.87	-1.30	[-2.80; 0.20]	2.1%
Saffi 2014	38	0.20	1.73	36	0.10	1.73	0.10	[-0.69; 0.89]	3.8%
Scain 2009	52	-0.30	1.41	52	0.20	1.40	-0.50	[-1.04; 0.04]	4.5%
Yin 2018	109	-0.31	0.05	75	-0.36	0.06	0.05	[0.03; 0.07]	5.5%
Hailu 2018	78	-2.88	4.28	64	-2.50	3.59	-0.38	[-1.67; 0.91]	2.5%
Prabhakaran 2018	332	-1.90	2.25	328	-1.80	2.40	-0.10	[-0.46; 0.26]	5.0%
Sriram 2011	60	-1.71	0.26	60	-0.72	0.40	-0.99	[-1.11; -0.87]	5.4%
Jarab 2012	77	-0.80	4.00	79	0.10	2.72	-0.90	[-1.98; 0.18]	3.0%
Wishah 2015	52	-1.70	1.39	54	-0.30	1.30	-1.40	[-1.91; -0.89]	4.6%
Ramli 2016	314	-0.10	1.94	278	0.10	2.02	-0.20	[-0.52; 0.12]	5.1%
Fairall 2016	180	0.00	2.40	229	-0.20	2.10	0.20	[-0.24; 0.64]	4.8%
Mash 2014	115	-0.50	2.17	140	-0.50	2.25	0.00	[-0.54; 0.54]	4.5%
Muchiri 2016	41	-1.00	1.86	41	-1.00	2.07	0.00	[-0.85; 0.85]	3.6%
Jayasuriya 2015	28	-2.80	1.99	25	-1.40	1.65	-1.40	[-2.38; -0.42]	3.2%
Guo 2019	75	-0.58	1.49	68	0.15	1.42	-0.73	[-1.21; -0.25]	4.7%
**Random effects model**	1743			1782			-0.45	[-0.78; -0.12]	69.9%
Heterogeneity: $I^2 = 95\%$, $\tau^2 = 0.3714$, $p < 0.01$									
**Above 15 months**									
Obreli 2011	97	-0.70	0.75	97	0.00	0.50	-0.70	[-0.88; -0.52]	5.3%
Ali 2016	575	-1.60	2.44	571	-1.20	2.68	-0.40	[-0.70; -0.10]	5.1%
**Random effects model**	672			668			-0.57	[-0.86; -0.28]	10.5%
Heterogeneity: $I^2 = 65\%$, $\tau^2 = 0.0293$, $p = 0.09$									
**Random effects model**	**2663**			**2682**			**-0.58**	**[-0.86; -0.30]**	**100.0%**
**Prediction interval**								**[-1.87; 0.71]**	

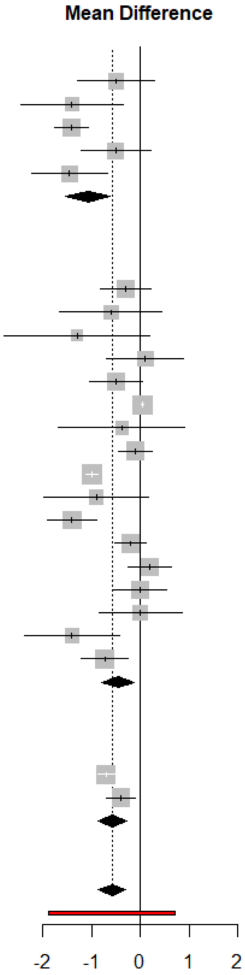

Heterogeneity: $I^2 = 95\%$, $\tau^2 = 0.3682$, $p < 0.01$
Residual heterogeneity: $I^2 = 94\%$, $p < 0.01$
Test for subgroup differences: $\chi^2_2 = 5.04$, df = 2 ($p = 0.08$)

Fig S5: Sub-group analysis by physician density (HbA1c)

Study	Experimental Total	Experimental Mean	Experimental SD	Control Total	Control Mean	Control SD	MD	95%-CI	Weight
**Upto 10**									
Depue 2013	104	-0.30	2.05	164	0.00	2.30	-0.30	[-0.83; 0.23]	4.6%
Hailu 2018	78	-2.88	4.28	64	-2.50	3.59	-0.38	[-1.67; 0.91]	2.5%
Prabhakaran 2018	332	-1.90	2.25	328	-1.80	2.40	-0.10	[-0.46; 0.26]	5.0%
Sriram 2011	60	-1.71	0.26	60	-0.72	0.40	-0.99	[-1.11; -0.87]	5.4%
Ali 2016	575	-1.60	2.44	571	-1.20	2.68	-0.40	[-0.70; -0.10]	5.1%
Azami 2018	71	-1.42	1.00	71	-0.01	1.13	-1.41	[-1.76; -1.06]	5.0%
Jahangard 2015	45	-1.00	1.55	40	-0.50	1.81	-0.50	[-1.22; 0.22]	4.0%
Nesari 2010	30	-1.87	1.33	30	-0.42	1.74	-1.45	[-2.23; -0.67]	3.8%
Fairall 2016	180	0.00	2.40	229	-0.20	2.10	0.20	[-0.24; 0.64]	4.8%
Mash 2014	115	-0.50	2.17	140	-0.50	2.25	0.00	[-0.54; 0.54]	4.5%
Muchiri 2016	41	-1.00	1.86	41	-1.00	2.07	0.00	[-0.85; 0.85]	3.6%
Jayasuriya 2015	28	-2.80	1.99	25	-1.40	1.65	-1.40	[-2.38; -0.42]	3.2%
Random effects model	1659			1763			-0.55	[-0.89; -0.21]	51.5%
Heterogeneity: $I^2 = 87\%$, $\tau^2 = 0.2638$, $p < 0.01$									
**11-15**									
Yin 2018	109	-0.31	0.05	75	-0.36	0.06	0.05	[0.03; 0.07]	5.5%
Goldhaber-Fiebert 2003	40	-1.80	2.30	35	-0.40	2.30	-1.40	[-2.44; -0.36]	3.1%
Ramli 2016	314	-0.10	1.94	278	0.10	2.02	-0.20	[-0.52; 0.12]	5.1%
Guo 2019	75	-0.58	1.49	68	0.15	1.42	-0.73	[-1.21; -0.25]	4.7%
Random effects model	538			456			-0.39	[-0.83; 0.06]	18.3%
Heterogeneity: $I^2 = 85\%$, $\tau^2 = 0.1472$, $p < 0.01$									
**Above 15**									
Moreira 2015	38	-1.30	2.26	39	-0.70	2.42	-0.60	[-1.65; 0.45]	3.1%
Mourao 2013	50	-0.60	3.80	50	0.70	3.87	-1.30	[-2.80; 0.20]	2.1%
Obreli 2011	97	-0.70	0.75	97	0.00	0.50	-0.70	[-0.88; -0.52]	5.3%
Saffi 2014	38	0.20	1.73	36	0.10	1.73	0.10	[-0.69; 0.89]	3.8%
De Souza 2017	62	-1.20	2.07	56	-0.70	2.33	-0.50	[-1.30; 0.30]	3.8%
Scain 2009	52	-0.30	1.41	52	0.20	1.40	-0.50	[-1.04; 0.04]	4.5%
Jarab 2012	77	-0.80	4.00	79	0.10	2.72	-0.90	[-1.98; 0.18]	3.0%
Wishah 2015	52	-1.70	1.39	54	-0.30	1.30	-1.40	[-1.91; -0.89]	4.6%
Random effects model	466			463			-0.71	[-1.01; -0.41]	30.2%
Heterogeneity: $I^2 = 44\%$, $\tau^2 = 0.0700$, $p = 0.08$									
**Random effects model**	**2663**			**2682**			**-0.58**	**[-0.86; -0.30]**	**100.0%**
**Prediction interval**								**[-1.87; 0.71]**	

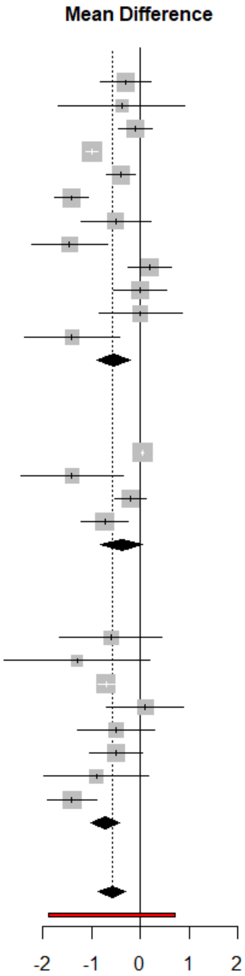

Heterogeneity: $I^2 = 95\%$, $\tau^2 = 0.3682$, $p < 0.01$
Residual heterogeneity: $I^2 = 82\%$, $p < 0.01$
Test for subgroup differences: $\chi^2_2 = 1.52$, df = 2 ($p = 0.47$)

Fig S6: Sub-group analysis by WHO Regions

Study	Experimental Total	Experimental Mean	Experimental SD	Control Total	Control Mean	Control SD	MD	95%-CI	Weight
**WPRO**									
Depue 2013	104	-0.30	2.05	164	0.00	2.30	-0.30	[-0.83; 0.23]	4.6%
Yin 2018	109	-0.31	0.05	75	-0.36	0.06	0.05	[0.03; 0.07]	5.5%
Ramli 2016	314	-0.10	1.94	278	0.10	2.02	-0.20	[-0.52; 0.12]	5.1%
Guo 2019	75	-0.58	1.49	68	0.15	1.42	-0.73	[-1.21; -0.25]	4.7%
**Random effects model**	602			585			-0.24	[-0.57; 0.09]	19.8%
Heterogeneity: $I^2 = 79\%$, $\tau^2 = 0.0828$, $p < 0.01$									
**PAHO**									
Moreira 2015	38	-1.30	2.26	39	-0.70	2.42	-0.60	[-1.65; 0.45]	3.1%
Mourao 2013	50	-0.60	3.80	50	0.70	3.87	-1.30	[-2.80; 0.20]	2.1%
Obreli 2011	97	-0.70	0.75	97	0.00	0.50	-0.70	[-0.88; -0.52]	5.3%
Saffi 2014	38	0.20	1.73	36	0.10	1.73	0.10	[-0.69; 0.89]	3.8%
De Souza 2017	62	-1.20	2.07	56	-0.70	2.33	-0.50	[-1.30; 0.30]	3.8%
Scain 2009	52	-0.30	1.41	52	0.20	1.40	-0.50	[-1.04; 0.04]	4.5%
Goldhaber-Fiebert 2003	40	-1.80	2.30	35	-0.40	2.30	-1.40	[-2.44; -0.36]	3.1%
**Random effects model**	377			365			-0.63	[-0.86; -0.41]	25.6%
Heterogeneity: $I^2 = 13\%$, $\tau^2 = 0.0150$, $p = 0.33$									
**AFRO**									
Hailu 2018	78	-2.88	4.28	64	-2.50	3.59	-0.38	[-1.67; 0.91]	2.5%
Fairall 2016	180	0.00	2.40	229	-0.20	2.10	0.20	[-0.24; 0.64]	4.8%
Mash 2014	115	-0.50	2.17	140	-0.50	2.25	0.00	[-0.54; 0.54]	4.5%
Muchiri 2016	41	-1.00	1.86	41	-1.00	2.07	0.00	[-0.85; 0.85]	3.6%
**Random effects model**	414			474			0.08	[-0.23; 0.39]	15.4%
Heterogeneity: $I^2 = 0\%$, $\tau^2 = 0$, $p = 0.83$									
**SEARO**									
Prabhakaran 2018	332	-1.90	2.25	328	-1.80	2.40	-0.10	[-0.46; 0.26]	5.0%
Sriram 2011	60	-1.71	0.26	60	-0.72	0.40	-0.99	[-1.11; -0.87]	5.4%
Ali 2016	575	-1.60	2.44	571	-1.20	2.68	-0.40	[-0.70; -0.10]	5.1%
Jayasuriya 2015	28	-2.80	1.99	25	-1.40	1.65	-1.40	[-2.38; -0.42]	3.2%
**Random effects model**	995			984			-0.64	[-1.16; -0.13]	18.8%
Heterogeneity: $I^2 = 91\%$, $\tau^2 = 0.2253$, $p < 0.01$									
**EMRO**									
Azami 2018	71	-1.42	1.00	71	-0.01	1.13	-1.41	[-1.76; -1.06]	5.0%
Jahangard 2015	45	-1.00	1.55	40	-0.50	1.81	-0.50	[-1.22; 0.22]	4.0%
Nesari 2010	30	-1.87	1.33	30	-0.42	1.74	-1.45	[-2.23; -0.67]	3.8%
Jarab 2012	77	-0.80	4.00	79	0.10	2.72	-0.90	[-1.98; 0.18]	3.0%
Wishah 2015	52	-1.70	1.39	54	-0.30	1.30	-1.40	[-1.91; -0.89]	4.6%
**Random effects model**	275			274			-1.23	[-1.56; -0.90]	20.4%
Heterogeneity: $I^2 = 32\%$, $\tau^2 = 0.0449$, $p = 0.21$									
**Random effects model**	**2663**			**2682**			**-0.58**	**[-0.86; -0.30]**	**100.0%**
**Prediction interval**								**[-1.87; 0.71]**	

Heterogeneity: $I^2 = 95\%$, $\tau^2 = 0.3682$, $p < 0.01$
Residual heterogeneity: $I^2 = 68\%$, $p < 0.01$
Test for subgroup differences: $\chi^2_4 = 35.72$, df = 4 ($p < 0.01$)

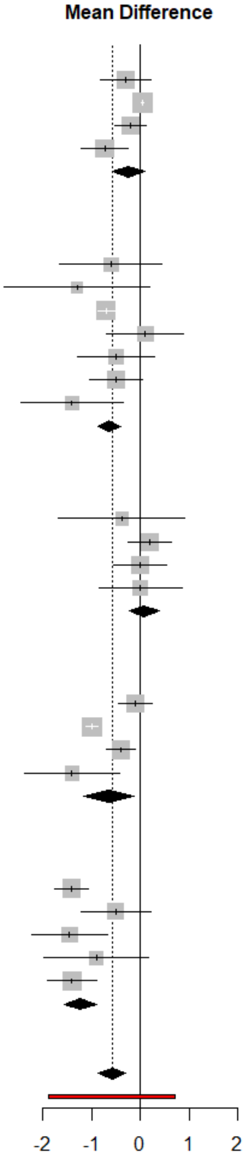

Fig S7: Funnel plot for FBS

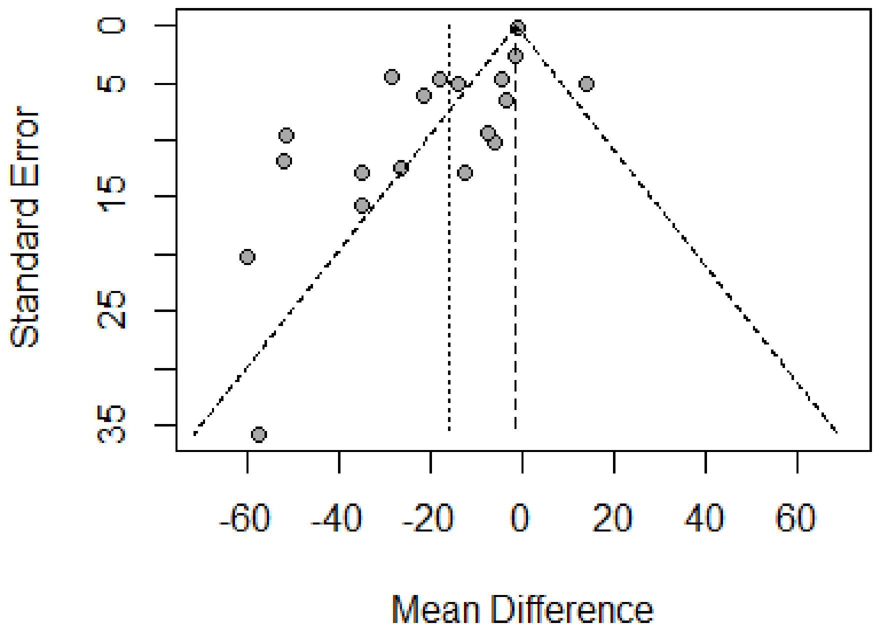

Fig S7.1: Contour enhanced funnel plot for FBS meta-analysis

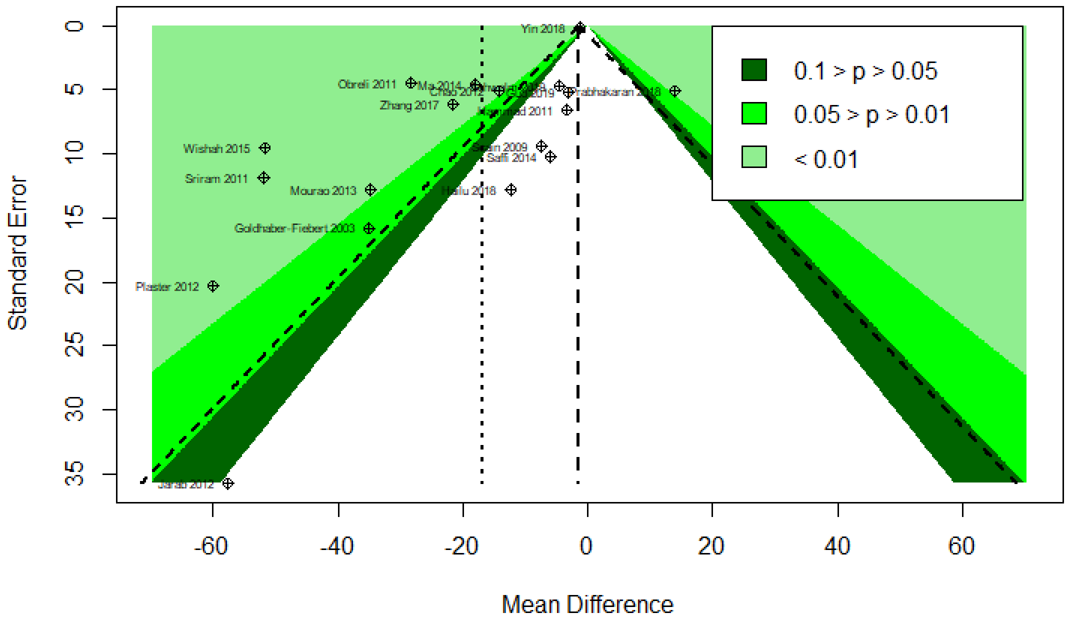

Fig S8: Sub-group analysis by task shared cadre (FBS) and type of intervention

Study	Experimental Total	Experimental Mean	Experimental SD	Control Total	Control Mean	Control SD	MD	95%-CI	Weight
**CHW: Non-Pharmacological Intervention**									
Chao 2012	112	-26.46	38.06	133	-12.24	41.88	-14.22	[-24.24; -4.20]	7.1%
Yin 2018	109	5.22	1.00	75	6.48	1.26	-1.26	[-1.60; -0.92]	8.1%
Random effects model	221			208			-6.73	[-19.28; 5.81]	15.2%
Heterogeneity: $I^2 = 84\%$, $\tau^2 = 70.9041$, $p = 0.48$									
**CHW: Pharmacological Intervention**									
Schwalm 2019	195	-0.72	47.59	222	3.78	48.31	-4.50	[-13.72; 4.72]	7.2%
Random effects model	195			222			-4.50	[-13.72; 4.72]	7.2%
Heterogeneity: not applicable									
**Dietitian: Non-Pharmacological Intervention**									
Goldhaber-Fiebert 2003	40	-19.00	55.00	35	16.00	78.00	-35.00	[-65.96; -4.04]	3.4%
Random effects model	40			35			-35.00	[-65.96; -4.04]	3.4%
Heterogeneity: not applicable									
**Nurse: Non-Pharmacological Intervention**									
Saffi 2014	38	-4.00	52.12	36	2.00	34.60	-6.00	[-26.06; 14.06]	5.1%
Scain 2009	52	-15.60	55.63	52	-8.20	38.67	-7.40	[-25.81; 11.01]	5.4%
Zhang 2017	100	-21.60	45.64	99	0.00	41.52	-21.60	[-33.72; -9.48]	6.7%
Hailu 2018	78	-27.00	88.32	64	-14.70	64.00	-12.30	[-37.40; 12.80]	4.2%
Prabhakaran 2018	332	-35.00	63.88	328	-49.00	67.45	14.00	[3.97; 24.03]	7.1%
Ma 2014	54	-7.02	27.67	52	10.98	19.14	-18.00	[-27.03; -8.97]	7.2%
Guo 2019	75	-3.60	31.05	68	-0.54	31.25	-3.06	[-13.28; 7.16]	7.0%
Random effects model	729			699			-7.46	[-18.44; 3.52]	42.8%
Heterogeneity: $I^2 = 79\%$, $\tau^2 = 162.1223$, $p = 0.01$									
**Pharmacist: Non-Pharmacological Intervention**									
Mourao 2013	50	-21.40	58.08	50	13.40	69.28	-34.80	[-59.86; -9.74]	4.2%
Plaster 2012	34	-68.00	82.93	29	-8.00	77.95	-60.00	[-99.77; -20.23]	2.5%
Sriram 2011	60	-88.32	68.56	60	-36.43	61.10	-51.89	[-75.13; -28.65]	4.5%
Random effects model	144			139			-46.47	[-62.13; -30.81]	11.2%
Heterogeneity: $I^2 = 0\%$, $\tau^2 = 0$, p = NA									
**Pharmacist: Pharmacological Intervention**									
Obreli 2011	97	-27.20	42.71	97	1.10	10.55	-28.30	[-37.05; -19.55]	7.3%
Hammad 2011	110	-13.30	47.60	89	-10.00	44.85	-3.30	[-16.18; 9.58]	6.5%
Jarab 2012	77	-41.40	273.96	79	16.20	154.98	-57.60	[-127.69; 12.49]	1.0%
Wishah 2015	52	-53.30	52.15	54	-1.60	46.02	-51.70	[-70.45; -32.95]	5.4%
Random effects model	336			319			-29.26	[-51.20; -7.32]	20.2%
Heterogeneity: $I^2 = 85\%$, $\tau^2 = 357.7266$, p = NA									
**Random effects model**	**1665**			**1622**			**-16.74**	**[-24.20; -9.29]**	**100.0%**
**Prediction interval**								**[-46.18; 12.70]**	

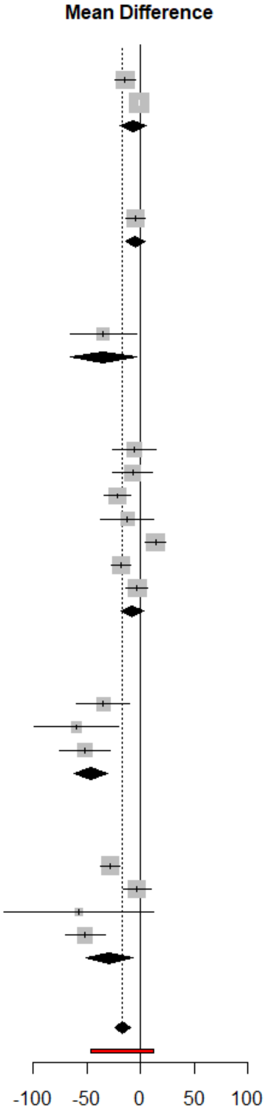

Heterogeneity: $I^2 = 88\%$, $\tau^2 = 178.3394$, $p < 0.01$

Residual heterogeneity: $I^2 = 79\%$, $p < 0.01$

Test for subgroup differences: $\chi^2_5 = 26.79$, df = 5 ($p < 0.01$)

Fig S9: Sub-group analysis by study population (FBS)

Study	Experimental Total	Experimental Mean	Experimental SD	Control Total	Control Mean	Control SD	MD	95%-CI	Weight
**Other conditions**									
Obreli 2011	97	-27.20	42.71	97	1.10	10.55	-28.30	[-37.05; -19.55]	7.3%
Saffi 2014	38	-4.00	52.12	36	2.00	34.60	-6.00	[-26.06; 14.06]	5.1%
Yin 2018	109	5.22	1.00	75	6.48	1.26	-1.26	[-1.60; -0.92]	8.1%
Zhang 2017	100	-21.60	45.64	99	0.00	41.52	-21.60	[-33.72; -9.48]	6.7%
Hammad 2011	110	-13.30	47.60	89	-10.00	44.85	-3.30	[-16.18; 9.58]	6.5%
Ma 2014	54	-7.02	27.67	52	10.98	19.14	-18.00	[-27.03; -8.97]	7.2%
Schwalm 2019	195	-0.72	47.59	222	3.78	48.31	-4.50	[-13.72; 4.72]	7.2%
Random effects model	703			670			-11.95	[-21.57; -2.33]	48.2%
Heterogeneity: $I^2 = 90\%$, $\tau^2 = 137.6134$, $p < 0.01$									
**T2DM**									
Mourao 2013	50	-21.40	58.08	50	13.40	69.28	-34.80	[-59.86; -9.74]	4.2%
Plaster 2012	34	-68.00	82.93	29	-8.00	77.95	-60.00	[-99.77; -20.23]	2.5%
Scain 2009	52	-15.60	55.63	52	-8.20	38.67	-7.40	[-25.81; 11.01]	5.4%
Chao 2012	112	-26.46	38.06	133	-12.24	41.88	-14.22	[-24.24; -4.20]	7.1%
Goldhaber-Fiebert 2003	40	-19.00	55.00	35	16.00	78.00	-35.00	[-65.96; -4.04]	3.4%
Hailu 2018	78	-27.00	88.32	64	-14.70	64.00	-12.30	[-37.40; 12.80]	4.2%
Prabhakaran 2018	332	-35.00	63.88	328	-49.00	67.45	14.00	[3.97; 24.03]	7.1%
Sriram 2011	60	-88.32	68.56	60	-36.43	61.10	-51.89	[-75.13; -28.65]	4.5%
Jarab 2012	77	-41.40	273.96	79	16.20	154.98	-57.60	[-127.69; 12.49]	1.0%
Wishah 2015	52	-53.30	52.15	54	-1.60	46.02	-51.70	[-70.45; -32.95]	5.4%
Guo 2019	75	-3.60	31.05	68	-0.54	31.25	-3.06	[-13.28; 7.16]	7.0%
Random effects model	962			952			-23.94	[-38.51; -9.37]	51.8%
Heterogeneity: $I^2 = 86\%$, $\tau^2 = 450.7733$, $p < 0.01$									
**Random effects model**	**1665**			**1622**			**-16.74**	**[-24.20; -9.29]**	**100.0%**
**Prediction interval**								**[-46.18; 12.70]**	

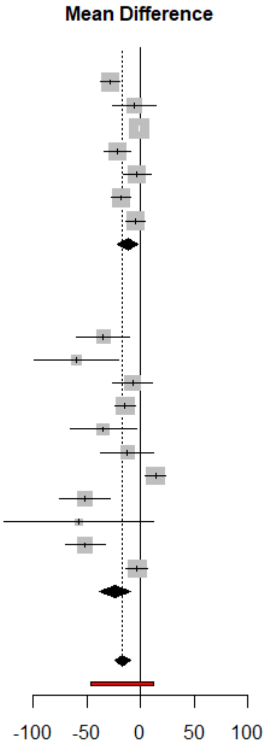

Heterogeneity: $I^2 = 88\%$, $\tau^2 = 178.3394$, $p < 0.01$
Residual heterogeneity: $I^2 = 88\%$, $p < 0.01$
Test for subgroup differences: $\chi^2_1 = 1.81$, df = 1 ($p = 0.18$)

Fig S9.1: Sub-group analysis by sample size (FBS)

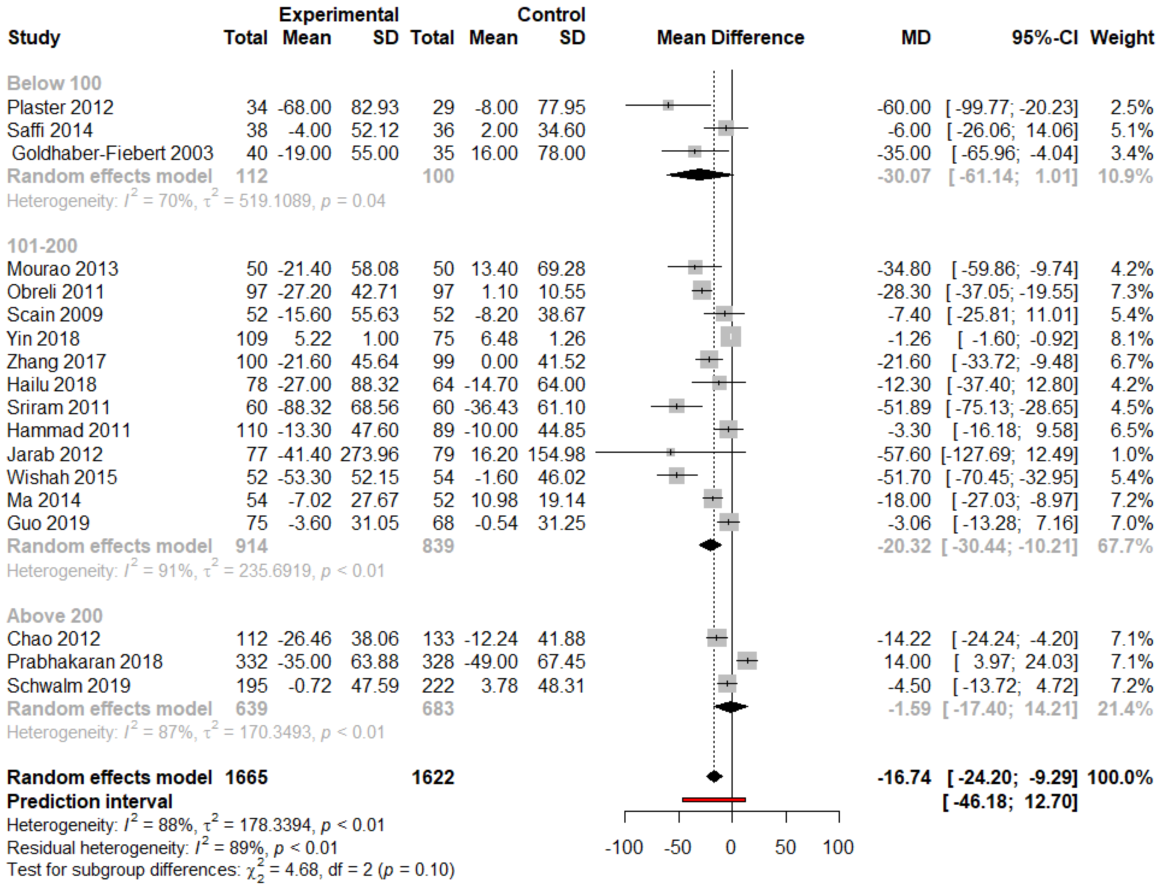

Fig S 10: Sub-group analysis by follow-up (FBS)

Study	Experimental Total	Experimental Mean	Experimental SD	Control Total	Control Mean	Control SD	MD	95%-CI	Weight
**Upto 5 months**									
Goldhaber-Fiebert 2003	40	-19.00	55.00	35	16.00	78.00	-35.00	[-65.96; -4.04]	3.4%
Random effects model	40			35			-35.00	[-65.96; -4.04]	3.4%
Heterogeneity: not applicable									
**6-15 months**									
Mourao 2013	50	-21.40	58.08	50	13.40	69.28	-34.80	[-59.86; -9.74]	4.2%
Plaster 2012	34	-68.00	82.93	29	-8.00	77.95	-60.00	[-99.77; -20.23]	2.5%
Saffi 2014	38	-4.00	52.12	36	2.00	34.60	-6.00	[-26.06; 14.06]	5.1%
Scain 2009	52	-15.60	55.63	52	-8.20	38.67	-7.40	[-25.81; 11.01]	5.4%
Yin 2018	109	5.22	1.00	75	6.48	1.26	-1.26	[-1.60; -0.92]	8.1%
Zhang 2017	100	-21.60	45.64	99	0.00	41.52	-21.60	[-33.72; -9.48]	6.7%
Hailu 2018	78	-27.00	88.32	64	-14.70	64.00	-12.30	[-37.40; 12.80]	4.2%
Prabhakaran 2018	332	-35.00	63.88	328	-49.00	67.45	14.00	[3.97; 24.03]	7.1%
Sriram 2011	60	-88.32	68.56	60	-36.43	61.10	-51.89	[-75.13; -28.65]	4.5%
Hammad 2011	110	-13.30	47.60	89	-10.00	44.85	-3.30	[-16.18; 9.58]	6.5%
Jarab 2012	77	-41.40	273.96	79	16.20	154.98	-57.60	[-127.69; 12.49]	1.0%
Wishah 2015	52	-53.30	52.15	54	-1.60	46.02	-51.70	[-70.45; -32.95]	5.4%
Ma 2014	54	-7.02	27.67	52	10.98	19.14	-18.00	[-27.03; -8.97]	7.2%
Schwalm 2019	195	-0.72	47.59	222	3.78	48.31	-4.50	[-13.72; 4.72]	7.2%
Guo 2019	75	-3.60	31.05	68	-0.54	31.25	-3.06	[-13.28; 7.16]	7.0%
Random effects model	1416			1357			-14.86	[-22.69; -7.03]	82.2%
Heterogeneity: $I^2 = 86\%$, $\tau^2 = 156.8373$, $p < 0.01$									
**Above 15 months**									
Obreli 2011	97	-27.20	42.71	97	1.10	10.55	-28.30	[-37.05; -19.55]	7.3%
Chao 2012	112	-26.46	38.06	133	-12.24	41.88	-14.22	[-24.24; -4.20]	7.1%
Random effects model	209			230			-21.48	[-35.27; -7.69]	14.4%
Heterogeneity: $I^2 = 77\%$, $\tau^2 = 76.0852$, $p = 0.04$									
**Random effects model**	**1665**			**1622**			**-16.74**	**[-24.20; -9.29]**	**100.0%**
**Prediction interval**								**[-46.18; 12.70]**	

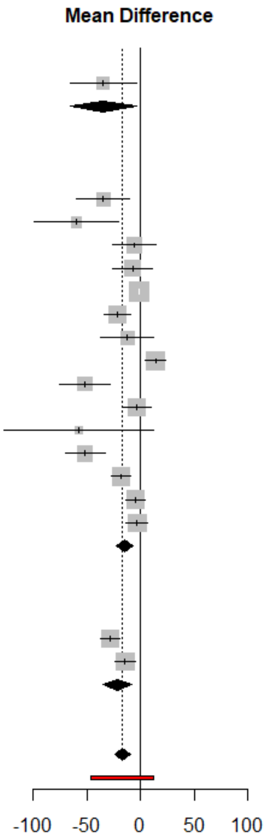

Heterogeneity: $I^2 = 88\%$, $\tau^2 = 178.3394$, $p < 0.01$
Residual heterogeneity: $I^2 = 85\%$, $p < 0.01$
Test for subgroup differences: $\chi^2_2 = 1.98$, df = 2 ($p = 0.37$)

Fig S11: Sub-group analysis by physician density (FBS)

Study	Experimental Total	Experimental Mean	Experimental SD	Control Total	Control Mean	Control SD	MD	95%-CI	Weight
**Upto 10**									
Hailu 2018	78	-27.00	88.32	64	-14.70	64.00	-12.30	[-37.40; 12.80]	4.2%
Prabhakaran 2018	332	-35.00	63.88	328	-49.00	67.45	14.00	[3.97; 24.03]	7.1%
Sriram 2011	60	-88.32	68.56	60	-36.43	61.10	-51.89	[-75.13; -28.65]	4.5%
**Random effects model**	**470**			**452**			**-15.82**	**[-56.60; 24.97]**	**15.8%**
Heterogeneity: $I^2 = 93\%$, $\tau^2 = 1191.6639$, $p < 0.01$									
**11-15**									
Chao 2012	112	-26.46	38.06	133	-12.24	41.88	-14.22	[-24.24; -4.20]	7.1%
Yin 2018	109	5.22	1.00	75	6.48	1.26	-1.26	[-1.60; -0.92]	8.1%
Zhang 2017	100	-21.60	45.64	99	0.00	41.52	-21.60	[-33.72; -9.48]	6.7%
Goldhaber-Fiebert 2003	40	-19.00	55.00	35	16.00	78.00	-35.00	[-65.96; -4.04]	3.4%
Ma 2014	54	-7.02	27.67	52	10.98	19.14	-18.00	[-27.03; -8.97]	7.2%
Schwalm 2019	195	-0.72	47.59	222	3.78	48.31	-4.50	[-13.72; 4.72]	7.2%
Guo 2019	75	-3.60	31.05	68	-0.54	31.25	-3.06	[-13.28; 7.16]	7.0%
**Random effects model**	**685**			**684**			**-10.96**	**[-18.61; -3.32]**	**46.8%**
Heterogeneity: $I^2 = 83\%$, $\tau^2 = 74.8640$, $p < 0.01$									
**Above 15**									
Mourao 2013	50	-21.40	58.08	50	13.40	69.28	-34.80	[-59.86; -9.74]	4.2%
Obreli 2011	97	-27.20	42.71	97	1.10	10.55	-28.30	[-37.05; -19.55]	7.3%
Plaster 2012	34	-68.00	82.93	29	-8.00	77.95	-60.00	[-99.77; -20.23]	2.5%
Saffi 2014	38	-4.00	52.12	36	2.00	34.60	-6.00	[-26.06; 14.06]	5.1%
Scain 2009	52	-15.60	55.63	52	-8.20	38.67	-7.40	[-25.81; 11.01]	5.4%
Hammad 2011	110	-13.30	47.60	89	-10.00	44.85	-3.30	[-16.18; 9.58]	6.5%
Jarab 2012	77	-41.40	273.96	79	16.20	154.98	-57.60	[-127.69; 12.49]	1.0%
Wishah 2015	52	-53.30	52.15	54	-1.60	46.02	-51.70	[-70.45; -32.95]	5.4%
**Random effects model**	**510**			**486**			**-25.60**	**[-39.76; -11.43]**	**37.4%**
Heterogeneity: $I^2 = 77\%$, $\tau^2 = 272.6549$, $p < 0.01$									
**Random effects model**	**1665**			**1622**			**-16.74**	**[-24.20; -9.29]**	**100.0%**
**Prediction interval**								**[-46.18; 12.70]**	

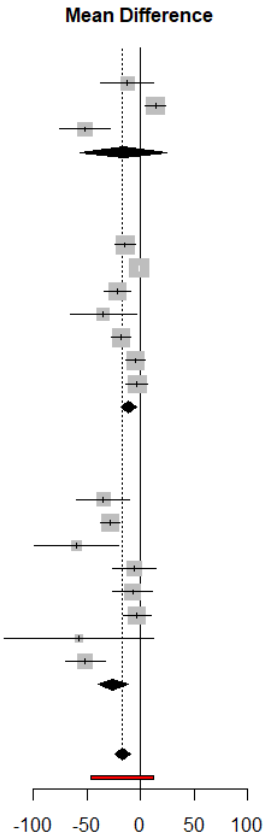

Heterogeneity: $I^2 = 88\%$, $\tau^2 = 178.3394$, $p < 0.01$

Residual heterogeneity: $I^2 = 84\%$, $p < 0.01$

Test for subgroup differences: $\chi^2_2 = 3.18$, df = 2 ($p = 0.20$)

Fig S12: Sub-group analysis by WHO geographical region

Study	Experimental Total	Experimental Mean	Experimental SD	Control Total	Control Mean	Control SD	MD	95%-CI	Weight
**PAHO**									
Mourao 2013	50	-21.40	58.08	50	13.40	69.28	-34.80	[-59.86; -9.74]	4.2%
Obreli 2011	97	-27.20	42.71	97	1.10	10.55	-28.30	[-37.05; -19.55]	7.3%
Plaster 2012	34	-68.00	82.93	29	-8.00	77.95	-60.00	[-99.77; -20.23]	2.5%
Saffi 2014	38	-4.00	52.12	36	2.00	34.60	-6.00	[-26.06; 14.06]	5.1%
Scain 2009	52	-15.60	55.63	52	-8.20	38.67	-7.40	[-25.81; 11.01]	5.4%
Goldhaber-Fiebert 2003	40	-19.00	55.00	35	16.00	78.00	-35.00	[-65.96; -4.04]	3.4%
Random effects model	311			299			-24.34	[-37.11; -11.56]	27.9%
Heterogeneity: $I^2 = 56\%$, $\tau^2 = 128.7090$, $p = 0.04$									
**WPRO**									
Chao 2012	112	-26.46	38.06	133	-12.24	41.88	-14.22	[-24.24; -4.20]	7.1%
Yin 2018	109	5.22	1.00	75	6.48	1.26	-1.26	[-1.60; -0.92]	8.1%
Zhang 2017	100	-21.60	45.64	99	0.00	41.52	-21.60	[-33.72; -9.48]	6.7%
Ma 2014	54	-7.02	27.67	52	10.98	19.14	-18.00	[-27.03; -8.97]	7.2%
Schwalm 2019	195	-0.72	47.59	222	3.78	48.31	-4.50	[-13.72; 4.72]	7.2%
Guo 2019	75	-3.60	31.05	68	-0.54	31.25	-3.06	[-13.28; 7.16]	7.0%
Random effects model	645			649			-9.72	[-17.22; -2.21]	43.4%
Heterogeneity: $I^2 = 84\%$, $\tau^2 = 67.2143$, $p < 0.01$									
**AFRO**									
Hailu 2018	78	-27.00	88.32	64	-14.70	64.00	-12.30	[-37.40; 12.80]	4.2%
Random effects model	78			64			-12.30	[-37.40; 12.80]	4.2%
Heterogeneity: not applicable									
**SEARO**									
Prabhakaran 2018	332	-35.00	63.88	328	-49.00	67.45	14.00	[3.97; 24.03]	7.1%
Sriram 2011	60	-88.32	68.56	60	-36.43	61.10	-51.89	[-75.13; -28.65]	4.5%
Random effects model	392			388			-18.08	[-82.63; 46.47]	11.6%
Heterogeneity: $I^2 = 96\%$, $\tau^2 = 2087.3905$, $p < 0.01$									
**EMRO**									
Hammad 2011	110	-13.30	47.60	89	-10.00	44.85	-3.30	[-16.18; 9.58]	6.5%
Jarab 2012	77	-41.40	273.96	79	16.20	154.98	-57.60	[-127.69; 12.49]	1.0%
Wishah 2015	52	-53.30	52.15	54	-1.60	46.02	-51.70	[-70.45; -32.95]	5.4%
Random effects model	239			222			-32.85	[-74.13; 8.43]	12.9%
Heterogeneity: $I^2 = 89\%$, $\tau^2 = 1030.9639$, $p < 0.01$									
**Random effects model**	**1665**			**1622**			**-16.74**	**[-24.20; -9.29]**	**100.0%**
**Prediction interval**								**[-46.18; 12.70]**	

Heterogeneity: $I^2 = 88\%$, $\tau^2 = 178.3394$, $p < 0.01$
Residual heterogeneity: $I^2 = 85\%$, $p < 0.01$
Test for subgroup differences: $\chi^2_4 = 4.60$, df = 4 ($p = 0.33$)

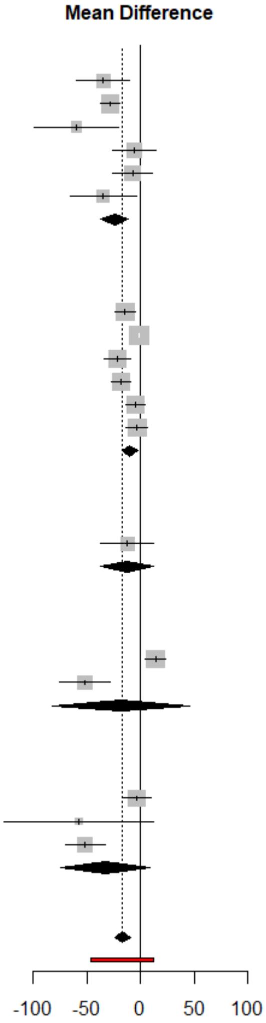

Box 1: Search strategy in PubMed, EMBASE and CINAHL

Diabetes: “Hyperlipidemias”[MeSH] OR “hyperlipidemias”[All Fields] OR “hyperlipidemia”[All Fields] OR “hyperlipidaemia”[All Fields] OR “hyperlipidaemias”[All Fields] OR “hyperlipemia”[All Fields] OR “hyperlipemias”[All Fields] OR “hyperlipaemia”[All Fields] OR “hyperlipaemias”[All Fields] OR “lipidemia”[All Fields] OR “lipidaemia”[All Fields] OR “high cholesterol”[All Fields] OR “hypercholesterolemia”[All Fields] OR “hypercholesterolemias”[All Fields] OR “hypercholesteremia”[All Fields] OR “hypercholesteremias”[All Fields] OR “hypercholesterolaemia”[All Fields] OR “hypercholesterolaemias”[All Fields] OR “hypercholesteraemia”[All Fields] OR “Diabetes”[All Fields] OR “diabetic”[All fields] OR "Diabetes Mellitus"[Mesh] OR “hyperglycemia” [All Fields] OR “blood sugar ” [All Fields] OR “Fasting blood sugar” [All Fields] OR “Glycosylated Haemoglobin” [All Fields] OR “Glycemic control” [All Fields] OR “proteinuria”[Mesh] OR “proteinuria”[All Fields] OR “Albuminuria”[All Fields] OR “Hemoglobinuria”[All Fields] OR "Kidney Failure, Chronic"[Mesh] OR “chronic kidney disease”[All Fields] OR “chronic renal disease”[All Fields] OR “chronic renal insufficiency”[All Fields] OR “CKD”[All Fields] OR “end-stage renal disease”[All Fields] OR “chronic kidney failure”[All Fields] OR “chronic renal failure”[All Fields] OR “chronic kidney diseases”[All Fields] OR “chronic renal diseases”[All Fields] OR “chronic renal insufficiencies”[All Fields] OR “end-stage renal diseases”[All Fields] OR “chronic kidney failures”[All Fields] OR “chronic renal failures”[All Fields] OR “stroke”[Mesh] OR “stroke”[All Fields] OR “strokes”[All Fields] OR “brain vascular accident”[All Fields] OR “brain vascular accidents”[All Fields] OR “apoplexy”[All Fields] OR "cerebrovascular accident”[All Fields] OR "cerebrovascular accidents”[All Fields] OR “cardiomyopathies”[Mesh] OR “cardiomyopathy”[All Fields] OR “cardiomyopathies”[All Fields] OR "myocardial disease”[All Fields] OR "myocardial diseases”[All Fields] OR “myocardiopathy”[All Fields] OR “myocardiopathies”[All Fields] OR “heart neoplasms”[Mesh] OR “heart neoplasm”[All Fields] OR “heart neoplasms”[All Fields] OR "cardiac tumor”[All Fields] OR "cardiac tumors”[All Fields] OR "myocardial tumor”[All Fields] OR "myocardial tumors”[All Fields] OR "cardiac carcinoma”[All Fields] OR "cardiac carcinomas”[All Fields] OR "heart cancer”[All Fields] OR "cardiac cancers”[All Fields] OR "cardiac cancer”[All Fields] OR "heart tumor”[All Fields] OR "heart tumors”[All Fields] OR “myocardial ischemia”[Mesh] OR “myocardial ischemia”[All Fields] OR “myocardial ischemias”[All Fields] OR "ischemic heart disease”[All Fields] OR "ischemic heart diseases”[All Fields] OR “myocardial ischaemia”[All Fields] OR “myocardial ischaemias”[All Fields] OR "ischaemic heart disease”[All Fields] OR "ischaemic heart diseases”[All Fields] OR “acute coronary syndrome”[All Fields] OR “acute coronary syndromes”[All Fields] OR “coronary disease”[All Fields] OR “coronary diseases”[All Fields] OR “coronary artery disease”[All Fields] OR “coronary artery diseases”[All Fields] OR “coronary arteriosclerosis”[All Fields] OR “Coronary atherosclerosis”[All Fields] OR “coronary stenosis”[All Fields] OR “coronary stenoses”[All Fields] OR “coronary restenosis”[All Fields] OR “coronary restenoses”[All Fields] OR “coronary heart disease”[All Fields] OR “coronary heart diseases”[All Fields] OR “coronary thrombosis”[All Fields] OR “coronary thromboses” OR “coronary occlusion”[All Fields] OR “coronary occlusions”[All Fields] OR “myocardial infarction”[All Fields] OR “myocardial infarctions”[All Fields] OR “heart attack”[All Fields] OR “heart attacks”[All Fields] OR “myocardial infarct”[All Fields] OR “myocardial infarcts”[All Fields] OR “heart arrest”[Mesh] OR “heart arrest”[All Fields] OR “heart arrests”[All Fields] OR “cardiac arrest”[All Fields] OR “cardiac arrests”[All Fields] OR “asystole”[All Fields] OR “asystoles”[All Fields] OR “cardiopulmonary arrest”[All Fields] OR “cardiopulmonary arrests”[All Fields] OR “heart failure”[Mesh] OR “heart failure”[All Fields] OR “heart failures”[All Fields] OR “cardiac failure”[All Fields] OR “cardiac failures”[All Fields] OR “myocardial failure”[All Fields] OR “myocardial failures”[All Fields] OR “heart decompensation”[All Fields] OR “hypertension”[Mesh] OR “hypertension”[All Fields] OR “hypertensions”[All Fields] OR “high blood pressure”[All Fields] OR “high blood pressures”[All Fields] OR “cardiovascular diseases”[Mesh] OR “cardiovascular disease”[All Fields] OR “cardiovascular diseases”[All Fields] OR "cardiovascular risk”[All Fields] OR "cardiovascular risks”[All Fields] OR “salt” [All Fields] OR “tobacco” [All Fields] OR “physical activity” [All Fields] OR “diet”[All Fields]

Task-Shifting: ((“Task”[All Fields] OR "tasks"[all fields]) AND ("shift"[All fields] OR "share"[All fields] OR "shifted"[all fields] OR "shifts"[all fields] OR "shifting"[all fields])) OR (shortage*[All Fields] AND ("physicians"[MeSH] OR “health personnel”[Mesh] OR "physicians"[All Fields] OR "doctors"[All Fields] OR "trained personnel"[All Fields] OR "health workforce"[All Fields] OR “health care workforce"[All Fields] OR “healthcare workforce"[All Fields] OR "health workers"[All Fields] OR "health care workers"[All Fields] OR “healthcare workers"[All Fields] OR "health care providers"[All Fields] OR “health providers"[All Fields] OR “healthcare providers"[All Fields])) OR ("nurse led"[All Fields] OR "primary health care nurse"[All Fields] OR "primary health care nurses"[All Fields] OR "primary health care nursing"[All Fields]) OR "nonphysician clinicians"[All Fields] OR "non-physician clinicians"[All Fields] OR “non physician health care workers"[All Fields] OR “nonphysician health care workers"[All Fields] OR “non physician health care worker"[All Fields] OR “nonphysician healthcare workers"[All Fields] OR “nonphysician health workers"[All Fields] OR “non physician health workers"[All Fields] OR ("role"[All Fields] AND ("nurse"[All Fields] OR "nurses"[all fields] OR "nursing"[all fields])) OR “community health aides”[All Fields] OR “community health centers”[mesh] OR “lay health workers"[All Fields] OR “lay health care workers"[All Fields] OR “lay health care worker"[All Fields] OR “community health workers"[All Fields] OR “community health care workers"[All Fields] OR “community healthcare workers"[All Fields] OR “community health center”[All Fields] OR “community Health centers”[all fields] OR “community health centre”[All fields] OR “community health centres”[All Fields] OR “extended scope practitioner”[all fields] OR “extended scope practitioners”[all fields] OR “extended scope practice”[all fields] OR “enhanced role”[all fields] OR “role enhancement”[all fields] OR ((“substitution”[All Fields] OR “substituted”[All Fields] OR “substitute”[All Fields] OR “substituting”[All Fields] OR “substitutes”[All Fields] OR “delegate”[All Fields] OR

“delegating”[All Fields] OR “delegates”[All Fields] OR “delegation”[All Fields] OR “delegated”[All Fields]) AND (“physicians”[mesh] OR “physician”[All Fields] OR “physicians”[All Fields] OR “doctor”[All Fields] OR “doctors”[All Fields]))

Countries: "developing countries"[all fields] OR "developing country"[all fields] OR "developing countries"[mesh] OR “medically underserved area” [mesh] OR “medically underserved area” [all fields] OR “medically underserved areas”[all fields] OR “low income countries”[all fields] OR “low income country”[all fields] OR “middle income countries”[all fields] OR “middle income country”[all fields] OR “global”[all fields] OR “resource poor”[all fields] OR “low resource”[all fields] OR “Africa”[Mesh] OR “Asia, Central”[Mesh] OR “Asia, Western”[Mesh] OR “Asia, Southeastern”[Mesh] OR “Indian Ocean Islands”[Mesh] OR “Central America”[Mesh] OR “South America”[Mesh] OR “Europe, Eastern”[Mesh] OR “Transcaucasia”[Mesh] OR “China”[Mesh] OR “Korea”[Mesh] OR “Mongolia”[Mesh] OR “Mexico”[Mesh] OR “Caribbean Region”[Mesh] OR “Pacific Islands”[Mesh] OR “Africa”[all fields] OR “Central Asia”[all fields] OR “western Asia”[all fields] OR “southeastern Asia”[all fields] OR “Indian Ocean Islands”[all fields] OR “Central America”[all fields] OR “South America”[all fields] OR “eastern Europe”[all fields] OR “Transcaucasia”[all fields] OR “Caribbean”[all fields] OR “Pacific Islands”[all fields] OR “Afghan”[all fields] OR “afghani”[all fields] OR “afghanistan”[all fields] OR “Bangladesh”[all fields] OR “bangladeshi”[all fields] OR “Benin”[all fields] OR “Beninese”[all fields] OR “Burkina Faso”[all fields] OR “Burkinabe”[all fields] OR “Burundi”[all fields] OR “burundian”[all fields] OR “Cambodia”[all fields] OR “cambodian”[all fields] OR “Central African Republic”[all fields] OR “central African”[all fields] OR “Chad”[all fields] OR “chadian”[all fields] OR “Comoros”[all fields] OR “comoran”[all fields] OR “Congo”[all fields] OR “congolese”[all fields] OR “Eritrea”[all fields] OR “eritrean”[all fields] OR “Ethiopia”[all fields] OR “ethiopian”[all fields] OR “Gambia”[all fields] OR “gambian”[all fields] OR “Guinea”[all fields] OR “guinean”[all fields] OR “Haiti”[all fields] OR “haitian”[all fields] OR “Kenya”[all fields] OR “Kenyan” OR “Korea”[all fields] OR “korean”[all fields] OR “Kyrgyz”[all fields] OR "kyrgyzstan"[all fields] OR “Liberia”[all fields] OR “liberian”[all fields] OR “Madagascar”[all fields] OR “malagasy”[all fields] OR “Malawi”[all fields] OR “malawian”[all fields] OR “mali”[all fields] OR “malian”[all fields] OR “mozambique”[all fields] OR “mozambican”[all fields] OR “Myanmar”[all fields] OR “myanmarese”[all fields] OR “burmese”[all fields] OR “Nepal”[all fields] OR “Nepalese”[all fields] OR “Niger”[all fields] OR “nigerian”[all fields] OR “Rwanda”[all fields] OR “rwandan”[all fields] OR “Sierra Leone”[all fields] OR “sierra leonean”[all fields] OR “Somalia”[all fields] OR “somalian”[all fields] OR “Tajikistan”[all fields] OR “tajik”[all fields] OR “tadzhik”[all fields] OR” Tanzania”[all fields] OR “tanzanian”[all fields] OR “Togo”[all fields] OR “togolese”[all fields] OR “Uganda”[all fields] OR “ugandan”[all fields] OR “Zimbabwe”[all fields] OR “zimbabwean”[all fields] OR “Angola”[all fields] OR “angolan”[all fields] OR “Armenia”[all fields] OR “armenian”[all fields] OR “Belize”[all fields] OR “belizean”[all fields] OR “Bhutan”[all fields] OR “bhutanese”[all fields] OR “Bolivia”[all fields] OR “bolivian”[all fields] OR “Cameroon”[all fields] OR “cameroonian”[all fields] OR “Cape Verde”[all fields] OR “cape verdian”[all fields] OR “cape verdean”[all fields] OR “Côte d'Ivoire” [all fields] OR "ivory coast"[all fields] “ivorian”[all fields] OR “Djibouti”[all fields] OR “Egypt”[all fields] OR “egyptian”[all fields] OR “El Salvador”[all fields] OR “salvadoran”[all fields] OR “Fiji”[all fields] OR “fijian”[all fields] OR “Georgia”[all fields] OR “georgian”[all fields] OR “Ghana”[all fields] OR “ghanaian”[all fields] OR “Guatemala”[all fields] OR “Guatemalan”[all fields] OR “Guyana”[all fields] OR “guyanese”[all fields] OR “Honduras” OR “honduran”[all fields] OR “Indonesia”[all fields] OR “indonesian”[all fields] OR “India”[all fields] OR “indian”[all fields] OR “Iraq”[all fields] OR “iraqi”[all fields] OR “Kiribati”[all fields] OR “Kosovo”[all fields] OR “kosovar”[all fields] OR “Laos”[all fields] OR “lao”[all fields] OR “laotian”[all fields] OR “Lesotho”[all fields] OR “Marshall Islands”[all fields] OR “marshallese”[all fields] OR “Mauritania”[all fields] OR “mauritanian”[all fields] OR “Micronesia”[all fields] OR “micronesian”[all fields] OR “Moldova”[all fields] OR “moldovan”[all fields] OR “Mongolia”[all fields] OR “mongolian”[all fields] OR “Morocco”[all fields] OR “moroccan”[all fields] OR “Nicaragua”[all fields] OR “nicaraguan”[all fields] OR “Nigeria”[all fields] OR “nigerian”[all fields] OR “Pakistan”[all fields] OR “pakistani”[all fields] OR “Papua New Guinea”[all fields] OR “papua new guinean”[all fields] OR “Paraguay”[all fields] OR “paraguayan”[all fields] OR “Philippines”[all fields] OR “filipino”[all fields] OR “Samoa”[all fields] OR “samoan”[all fields] OR “Sao Tome and Principe”[all fields] OR "São Tomé and Príncipe"[all fields] OR “Senegal”[all fields] OR “senegalese”[all fields] OR “Solomon Islands”[all fields] OR “Solomon islander”[all fields] OR “Sri Lanka”[all fields] OR “srilankan”[all fields] OR “Sudan”[all fields] OR “sudanese”[all fields] OR “Swazi”[all fields] OR “swaziland”[all fields] OR “Syria”[all fields] OR “syrian”[all fields] OR “east Timor”[all fields] OR “east timorese”[all fields] OR “Tonga”[all fields] OR “tongan”[all fields] OR “Turkmenistan”[all fields] OR “turkmen”[all fields] OR “Tuvalu”[all fields] OR “tuvaluan”[all fields] OR “Ukraine”[all fields] OR “ukrainian”[all fields] OR “Uzbekistan”[all fields] OR “uzbek”[all fields] OR “Vanuatu”[all fields] OR “Vietnam”[all fields] OR “vietnamese”[all fields] OR “West Bank”[all fields] OR

EMBASE

**CVD**	exp hyperlipidemia/ or hyperlipid?emia$1.mp. or hyperlip?emia$1.mp. or lipid?emia$1.mp. or high cholesterol.mp. or hypercholesterol?emia$1.mp. or hypercholester?emia$1.mp. or exp Diabetes mellitus/or diabetes.mp. OR exp diabetic angiopathy/ or diabetic.mp. or exp proteinuria/ or

	proteinuria$1.mp. or albuminuria$1.mp. or hemoglobinuria$1.mp. or exp chronic kidney disease/ or chronic kidney disease$1.mp. or chronic renal disease$1.mp. or chronic renal insufficienc$.mp. OR CKD.mp. OR endstage renal disease$1.mp. or chronic kidney failure$1.mp. or chronic renal failure$1.mp. or exp stroke/ or stroke$1.mp. or brain vascular accident$1.mp. or apoplexy.mp. or cerebrovascular accident$1.mp. or exp myocardial disease/ or cardiomyopath$.mp. or myocardial disease$1.mp. or myocardiopath$.mp. or heart muscle isch?emia$1.mp. or myocardial isch?emia$1.mp. or isch?emic heart disease$1.mp. or acute coronary syndrome$1.mp. or coronary disease$1.mp. or coronary artery disease$1.mp. or coronary arterioscleros$.mp. or coronary atheroscleros$.mp. or coronary stenos$.mp. or coronary restenos$.mp. or coronary heart disease$1.mp. or coronary thrombos$.mp. or coronary occlusion$1.mp. or myocardialinfarct$.mp. or heart attack$1.mp. or exp heart tumor/ or heart neoplasm$1.mp. or cardiac tumor$1.mp. or myocardial tumor$1.mp. or cardiac carcinoma$1.mp. or heart cancer$1.mp. or cardiac cancer$1.mp. or heart tumor$1.mp. or exp heart failure/ or heart arrest$1.mp. or cardiac arrest$1.mp. or asystole$1.mp. or cardiopulmonary arrest$1.mp. or heart failure$1.mp. or cardiac failure$1.mp. or myocardial failure$1.mp. or heart decompensation$1.mp. or exp hypertension/ or hypertension$1.mp. or high blood pressure$1.mp. or exp cardiovascular disease/ or exp cardiovascular risk/ or (cardiovascular ADJ5 disease$1).mp. or (cardiovascular ADJ5 risk$1).mp.
**Task shifting**	exp personnel shortage/ or (shortage$1 ADJ5 doctor$1).mp. or (shortage$1 ADJ5 physician$1).mp. or (shortage$1 ADJ5 trained ADJ5 personnel).mp. or (shortage$1 ADJ5 health ADJ5 workforce).mp. or (shortage$1 ADJ5 health ADJ5 worker$1).mp. or (shortage$1 ADJ5 health ADJ5 provider$1).mp. or (task$1 ADJ5 shift$).mp. or nurse led.mp. or non$1physician clinician$1.mp. or non$1physician health$ worker$1.mp. or primary health care nurs$.mp. or (role ADJ5 nurs$).mp. or exp community health nursing/ or exp health auxiliary/ or community health$ worker$1.mp. or community health cent$.mp. or lay health$ worker$1.mp. or community health$ aide$1.mp. or (community ADJ2 health ADJ5 worker$1).mp. or extended scope practi$.mp. or (role ADJ3 enhance$).mp. or (substitute$ ADJ10 physician$1).mp. or (substitute$ ADJ10 doctor$1).mp. or (substitute$ ADJ10 nurse$1).mp. or (delegat$ ADJ10 physician$1).mp. or (delegat$ ADJ10 doctor$1).mp. or (delegat$ ADJ10 nurse$1).mp.
**Low-and Middle-income countries:**	exp developing country/ or exp medically underserved/ or developing countr$.mp. or medically underserved area$1.mp. or low income countr$.mp. or middle income country.mp. or low resource.mp. or resource poor.mp. or global.mp. or exp Africa/ or exp "South and Central America"/or exp asia/ or exp Caribbean islands/ or exp pacific islands/ or exp eastern Europe/ or exp Indian Ocean/or south america$1.mp. or Africa$1.mp. or Caribbean.mp. or central America$1.mp. or south America$1.mp. or eastern Europe$1.mp. or pacific island$.mp. or Indian ocean island$.mp. or asia.mp. or Afghan$.mp. or Bangladesh$1.mp. or Benin$.mp. or Burkina Faso.mp. or Burkinabe.mp. or Burundi$.mp. or Cambodia$1.mp. or Central African.mp. or Chad$.mp. or Comor$.mp. or Congo$.mp. or Eritrea$1.mp. or Ethiopia$1.mp. or Gambia$1.mp. or Guinea$1.mp. or Haiti$.mp. or Kenya$1.mp. or Korea$1.mp. or exp North Korea/ or Kyrgyz$.mp. or Liberia$1.mp. or Madagascar.mp. or Malagasy.mp. or Malawi$.mp. or mali$.mp. or mozambi$.mp. or Myanmar$.mp. **or** Nepal$.mp. or Niger$.mp. or Rwanda$1.mp. or Sierra Leone$.mp. or Somalia$1.mp. or Tajik$.mp. or Tanzania$1.mp. or Togo$.mp. or Uganda$1.mp. or Zimbabwe$.mp. or Angola$1.mp. or Armenia$1.mp. or Beliz$.mp. or Bhutan$.mp. or Bolivia$1.mp. or Cameroon$.mp. or Cape Verde$.mp. or Congo$.mp. or "Côte d'Ivoire".mp. or Ivory Coast.mp. or Ivorian.mp. or Djibouti.mp. or Egypt$.mp. or El Salvador.mp. or Salvadoran.mp. or Fiji$.mp. or Georgia$1.mp. or Ghana$.mp. or Guatemala$1.mp. or Guyan$.mp. or Hondura$.mp. or Indonesia$1.mp. or India$1.mp. or Iraq$1.mp. or Kiribati.mp. or Kosov$.mp. or Lao$.mp. or Lesotho.mp. or Marshall Islands.mp. or Marshallese.mp. or Mauritania$1.mp. or Micronesia$1.mp. or Moldov$.mp. or Mongolia$1.mp. or Morocc$.mp. or Nicaragua$1.mp. or Nigeria$1.mp. or Pakistan$1.mp. or Papua New Guinea$1.mp. or Paraguay$.mp. or Philippines.mp. or Filipino.mp. or Samoa$1.mp. or sao tome$.mp. or Senegal$.mp. or Solomon Island$.mp. or sri lanka$1.mp. or Sudan$.mp. or Swazi$.mp. or Syria$1.mp. or Timor$.mp. or Tonga$1.mp. or Turkmen$.mp. or Tuvalu$.mp. or Ukrain$.mp. or Uzbek$.mp. or Vanuat$1.mp. or Vietnam$.mp. or West Bank.mp. or Gaza.mp. or Yemen$.mp. or Zambia$1.mp. or Albania$1.mp. or Algeria$1.mp. or "Antigua and Barbuda".mp. or antiguan.mp. or barbudan.mp. or Argentin$.mp. or Azerbaijan$1.mp. or Belarus$.mp. or Bosnia$1.mp. or Botswana.mp. or Brazil$.mp. or Bulgaria$1.mp. or Chile$.mp. or China.mp. or Chinese.mp. or Colombia$1.mp. or Costa Rica$1.mp. or

	Cuba$1.mp. or Dominica$1.mp. or Ecuador$.mp. or Gabon$.mp. or Grenad$.mp. or Iran$.mp. or Jamaica$1.mp. or Jordan$.mp. or Kazakhstan$1.mp. or Latvia$1.mp. or Leban$.mp. or Libya$1.mp. or Lithuania$1.mp. or Macedonia$1.mp. or Malaysia$1.mp. or Maldiv$.mp. or mauriti$.mp. or Mexic$.mp. or Montenegr$.mp. or Namibia$1.mp. or Palau$.mp. or Panama$.mp. or Peru$.mp. or Romania$1.mp. or Russia$1.mp. or Serbia$1.mp. or Seychell$.mp. or South Africa$1.mp. or Saint Kitts.mp. or Saint Lucia.mp. or Saint Vincent.mp. or Suriname$1.mp. or Thai$.mp. or Tunisia$1.mp. or Turk$.mp. or Uruguay$.mp. or Venezuala$1.mp.

CINAHL

S4	S1 AND S2 AND S3
S3	(MH "developing countries+") OR (MH "medically underserved area+") OR (TX "developing countr*") OR (TX "medically underserved area#") OR (TX "low income countr *") OR (TX "middle income countr*") OR (MH "Africa+") OR (TX Africa#) OR (TX Caribbean) OR (MH "west indies+") OR (TX "central America#") OR (MH "Central America+") OR (TX "south America#") OR (MH "south America+") OR (TX global) OR (TX "low resource") OR (TX "resource poor") OR (TX "central asia#") OR (MH "asia, central+") OR (TX "southeastern asia#") OR (MH "asia, southeastern+") OR (TX "western asia#") OR (MH "asia, western+") OR (TX "Indian ocean islands") OR (MH "Indian ocean islands+") OR (TX "eastern Europe*") OR (MH "europe, eastern") OR (TX Transcaucasia#) OR (TX "pacific islands")OR (MH "pacific islands+") OR (TX Afghan*) OR (TX Bangladesh#) OR (TX Benin*) OR (TX "Burkina Faso") OR (TX burkinabe) OR (TX Burundi*) OR (TX Cambodia#) OR (TX "Central African") OR (TX Chad*) OR (TX Comor*) OR (TX Congo*) OR (TX Eritrea#) OR (TX Ethiopia#) OR (TX Gambia#) OR (TX Guinea#) OR (TX Haiti*) OR (TX Kenya#) OR (TX Korea#) OR (TX Kyrgyz*) OR (TX Liberia#) OR (TX Madagascar) OR (TX malagasy) OR (TX Malawi*) OR (TX mali*) OR (TX mozambi*) OR (TX Myanmar*) OR (TX Nepal*) OR (TX Niger*) OR (TX Rwanda#) OR (TX "Sierra Leon*") OR (TX Somalia#) OR (TX Tajik*) OR (TX tadzhik) OR (TX Tanzania#) OR (TX Togo*) OR (TX Uganda#) OR (TX Zimbabwe*) OR (TX Angola#) OR (TX Armenia#) OR (TX Belize*) OR (TX Bhutan*) OR (TX Bolivia#) OR (TX Cameroon*) OR (TX Cape Verd*) OR (TX Congo*) OR (TX "Côte d'Ivoire") OR (TX "ivory coast") OR (TX ivorian) OR (TX Djibouti) OR (TX Egypt#) OR (TX "El Salvador") OR (TX "Salvadoran") OR (TX Fiji*) OR (TX Georgia#) OR (TX Ghana*) OR (TX Guatemala#) OR (TX Guyan*) OR (TX Hondura#) OR (TX Indonesia#) OR (TX India#) OR (TX Iraq#) OR (TX Kiribati) OR (TX Kosov*) OR (TX Lao*) OR (TX Lesotho) OR (TX ("Marshall Islands")) OR (TX marshallese) OR (TX Mauritania#) OR (TX Micronesia#) OR (TX Moldova#) OR (TX Mongolia#) OR (TX Morocc*) OR (TX Nicaragua#) OR (TX Nigeria#) OR (TX Pakistan#) OR (TX "Papua New Guinea*") OR (TX Paraguay*) OR (TX Philippines) OR (TX Filipino) OR (TX Samoa#) OR (TX "Sao Tome*") OR (TX Senegal*) OR (TX "Solomon Island*") OR (TX "sri lanka#") OR (TX Sudan*) OR (TX Swazi*) OR (TX Syria#) OR (TX Timor*) OR (TX Tonga#) OR (TX Turkmen*) OR (TX Tuvalu*) OR (TX Ukrain*) OR (TX Uzbek*) OR (TX Vanuat*) OR (TX Vietnam*) OR (TX ("West Bank")) OR (TX Gaza) OR (TX Yemen*) OR (TX Zambia#) OR (TX Albania#) OR (TX Algeria#) OR (TX "Antigua and Barbuda") OR (TX antiguan) OR (TX barbudan) OR (TX Argentin*) OR (TX Azerbaijani*) OR (TX Belarus*) OR (TX Bosnia#) OR (TX Botswana) OR (TX Brazil*) OR (TX Bulgaria#) OR (TX Chile*) OR (TX China) OR (TX Chinese) OR (MH "China+") OR (TX Colombia#) OR (TX "Costa Rica#") OR (TX Cuba#) OR (TX Dominica#) OR (TX Ecuador*) OR (TX Gabon*) OR (TX Grenad*) OR (TX Iran*) OR (TX Jamaica#) OR (TX Jordan*) OR (TX Kazakhstan#) OR (TX Latvia#) OR (TX Leban*) OR (TX Libya#) OR (TX Lithuania#) OR (TX Macedonia#) OR (TX Malaysia#) OR (TX Maldiv*) OR (TX mauriti*) OR (TX Mexic*) OR (TX Montenegr*) OR (TX Namibia#) OR (TX Palau*) OR (TX Panama*) OR (TX Peru*) OR (TX Romania#) OR (TX Russia#) OR (TX Serbia#) OR (TX Seychell*) OR (TX "South Africa#") OR (TX "Saint Kitts") OR (TX "Saint Lucia") OR (TX "Saint Vincent") OR (TX Suriname#) OR (TX Thai*) OR (TX Tunisia#) OR (TX Turk*) OR (TX Uruguay*) OR (TX Venezuala#)
S2	(MH "Personnel Shortage+") OR (TX shortage# N5 doctor#) OR (TX shortage# N5 physician#) OR (TX shortage# N5 "trained personnel") OR (TXshortage# N5 "health* * workforce") OR (TX shortage# N5 "health* * worker#") OR (TX shortage# N5 "health* * provider#") OR (TX task# N5 shift*) OR (TX "nurse led") OR (TX "non*physician clinicians") OR (TX "non*physician health* * worker#") OR (TX "primary health* * nurs*") OR (TX role N5 nurs*) OR (MH "community health workers+") OR (MH "community health centers+") OR (TX "lay health* * worker#") OR (TX community N2 "health* * aide#") OR (TX community N2 "health* * worker#") OR (TX community N2 "health* * cent*") OR (TX "extended scope practi*") OR (TX role N3 enhance*) OR (TX substitut* N10 physician#) OR (TX substitut* N10 doctor#) OR (TX substitute* N10 nurse#) OR (TX delegat* N10 physician#) OR (TX delegat * N10 doctor#) OR (TX delegat * N10 nurse#)
S1	(MH "cardiovascular diseases+") OR (TX cardiovascular N5 disease#) OR (MH "cardiovascular risk factors+") OR (TX cardiovascular N5 risk#) OR (MH "Hyperlipidemia+") OR (TX hyperlipid#emia#) OR (TX hyperlip#emia#) OR (TX

	lipid#emia#) OR (TX "high cholesterol") OR (TX hypercholesterol#emia#) OR (TX hypercholester#emia#) OR (TX diabetes) OR (TX diabetic) OR (MH "Diabetes Mellitus+") OR (MH "proteinuria+") OR (TX proteinuria#) OR (TX albuminuria#) OR (TX hemoglobinuria#) OR (MH "Kidney Failure, Chronic") OR (TX "chronic kidney disease#") OR (TX "chronic renal disease#") OR (TX "chronic renal insufficienc*") OR (TX "CKD") OR (TX "end-stage renal disease#") OR (TX "chronic kidney failure#") OR (TX "chronic renal failure#") OR (TX stroke#) OR (TX "brain vascular accident#") OR (TX apoplexy*) OR (TX "cerebrovascular accident#") OR (TX cardiomyopath*) OR (TX "myocardial disease#") OR (TX myocardiopath*) OR (TX "heart neoplasm#") OR (TX "cardiac tumor#") OR (TX "myocardial tumor#") OR (TX "cardiac carcinoma#") OR (TX "heart cancer#") OR (TX "cardiac cancer#") OR (TX "heart tumor#") OR (TX "myocardial isch#emia#") OR (TX "isch#emic heart disease#") OR (TX "acute coronary syndrome#") OR (TX "coronary disease#") OR (TX "coronary artery disease#") OR (TX "coronary arterioscleros*") OR (TX "coronary atheroscleros*") OR (TX "coronary stenos*") OR (TX "coronary restenos*") OR (TX "coronary heart disease*") OR (TX "coronary thrombos*") OR (TX "coronary occlusion#") OR (TX "myocardial infarct*") OR (TX "heart attack#") OR (TX "heart arrest#") OR (TX "cardiac arrest#") OR (TX asystole#) OR (TX "cardiopulmonary arrest#") OR (TX "heart failure#") OR (TX "cardiac failure#") OR (TX "myocardial failure#") OR (TX "heart decompensation#") OR (TX hypertension#) OR (TX "high blood pressure#")
